# Supplementary material for: Gene-informed decomposition model predicts lower soil carbon loss due to persistent microbial adaptation to warming
Source: Nat Commun. 2020 Sep 29;11:4897. doi: 10.1038/s41467-020-18706-z (PMC7524716; doi:10.1038/s41467-020-18706-z)
Supplement: Supplementary file 1 — Supplementary Information [file 41467_2020_18706_MOESM1_ESM.pdf]

Supplementary Information

Gene-informed decomposition model predicts lower soil carbon loss due to persistent microbial adaptation to warming

Guo et al.

Includes
Supplementary Tables (1-11)
Supplementary Figures (1-16)

Supplementary Table 1. Summary of sequence and GeoChip statistics. The microbial samples from each year were analyzed with various molecular approaches.

Sequencing/ GeoChip	Targets	Numbers of samples analyzed	Total base pairs (bp)	Average No. of reads/probes per sample	OTUs/ genes
16S rRNA gene amplicon sequencing	Bacteria + archaea	56	0.74G	51,415±2,696	26,158
ITS amplicon sequencing	Fungi	56	0.43G	3,1203±4,017	5,336
Shotgun sequencing	Functional genes	56	0.96T	127.83±2.89M	98,682
GeoChip	Functional genes	56	NA	35,425±468	35,425

Supplementary Table 2. The correlations between the structure of each functional gene group involved soil C decomposition and N cycling processes and each environmental attribute revealed by CCA analysis. Significance is adjusted by false discovery rate (FDR) and the p values are shown here.

		**GeoChip**			**Metagenome based EcoFUN-MAP**			**Metagenomic sequencing**		
	Attributes+	R_h	R_t	Q_{10}	R_h	R_t	Q_{10}	R_h	R_t	Q_{10}
	Carbon cycling	<0.01	<0.01	0.05	0.02	<0.01	0.07	0.08	<0.01	0.02
	Carbon degradation	<0.01	<0.01	0.03	0.10	<0.01	0.10	0.04	0.07	0.03
**C degradation**	Starch	<0.01	<0.01	0.02	0.20	0.04	0.51	0.02	0.09	0.30
	Pectin	<0.01	<0.01	0.03	0.90	0.97	0.67	0.40	0.47	0.01
	Hemicellulose	<0.01	0.02	0.31	0.27	0.02	0.12	0.18	0.07	0.02
	Cellulose	<0.01	<0.01	0.02	0.38	0.13	0.24	0.09	0.09	0.47
	Chitin	<0.01	<0.01	0.03	0.67	0.09	0.36	<0.01	0.07	0.80
	Other	<0.01	<0.01	0.03	0.03	<0.01	0.08	0.08	0.03	0.07
	Vanillin/lignin	<0.01	<0.01	0.02	0.09	0.07	<0.01	0.30	0.04	0.01
**N cycling**	Ammonification	<0.01	<0.01	0.03	0.02	<0.01	0.04	<0.01	<0.01	0.03
	Anammox	0.12	0.60	0.96	0.85	0.84	0.85	<0.01	<0.01	0.18
	Assimilatory N reduction	<0.01	<0.01	0.03	0.10	<0.01	0.01	0.30	<0.01	0.02
	Denitrification	<0.01	0.03	0.28	0.33	0.36	0.44	0.26	0.04	0.12
	Dissimilatory N reduction	0.22	0.08	0.09	0.48	0.03	0.49	0.71	0.59	0.02
	Nitrification	<0.01	<0.01	0.01	0.52	0.52	0.50	<0.01	<0.01	0.18
	Nitrogen fixation	<0.01	<0.01	0.02	0.37	0.62	0.91	0.43	0.39	0.16
**P utilization**	P utilization	<0.01	<0.01	0.03	0.04	0.01	0.03	<0.01	<0.01	0.04
**S metabolism**	Adenylylsulfate reductase	<0.01	<0.01	0.06	0.33	0.36	0.44	0.83	0.01	0.05
	Sulfur assimilation	<0.01	<0.01	0.05	<0.01	<0.01	0.18	0.76	0.13	0.34
	Sulfite reduction	<0.01	<0.01	<0.01	0.48	0.11	0.81	0.01	<0.01	<0.01
	Sulfide oxidation	<0.01	<0.01	0.05	0.02	<0.01	0.42	0.73	0.12	0.43

+ Abbreviation of environmental attributes: R_h, heterotrophic respiration; R_t, soil total respiration; Q_{10}, temperature sensitivity of heterotrophic respiration.

Supplementary Table 3. The correlations between the structure of each functional gene group involved soil C decomposition and N cycling processes and each environmental attribute revealed by Mantel test. Significance is adjusted by false discovery rate (FDR) and the p values are shown here.

		GeoChip			Metagenome based EcoFUN-MAP			Metagenomic sequencing		
	Attributes+	R_h	R_t	Q_{10}	R_h	R_t	Q_{10}	R_h	R_t	Q_{10}
	Carbon cycling	0.01	<0.01	0.01	0.23	0.08	0.42	0.01	0.04	0.1
	Carbon degradation	<0.01	<0.01	0.01	0.13	0.01	0.16	0.01	0.07	0.1
**C degradation**	Starch	<0.01	<0.01	0.01	0.04	<0.01	0.27	0.05	0.07	0.46
	Pectin	0.02	<0.01	0.02	0.06	<0.01	0.03	0.73	0.16	0.11
	Hemicellulose	<0.01	<0.01	0.03	0.07	0.02	0.07	0.03	0.02	0.14
	Cellulose	<0.01	<0.01	0.02	0.16	0.03	0.05	0.16	0.19	0.12
	Chitin	<0.01	<0.01	0.01	0.38	<0.01	0.34	0.28	0.02	0.23
	Other	<0.01	<0.01	0.01	0.29	0.12	0.36	0.08	0.04	0.06
	Vanillin/lignin	<0.01	<0.01	0.01	0.15	0.04	0.12	0.28	0.47	0.3
**N cycling**	Ammonification	0.04	<0.01	0.01	0.11	0.12	0.29	0.03	0.02	<0.01
	Anammox	0.15	0.73	0.72	0.38	0.67	0.74	0.01	<0.01	0.06
	Assimilatory N reduction	0.01	<0.01	0.01	0.18	<0.01	0.07	0.01	0.01	0.1
	Denitrification	<0.01	<0.01	0.01	0.37	0.37	0.14	0.21	0.26	0.65
	Dissimilatory N reduction	0.17	0.04	0.03	0.38	0.03	0.77	0.15	0.79	0.69
	Nitrification	0.11	0.01	0.01	0.86	0.66	0.29	<0.01	<0.01	0.06
	Nitrogen fixation	<0.01	<0.01	<0.01	0.76	0.7	0.53	0.32	0.12	0.01
**P utilization**	P utilization	<0.01	<0.01	0.01	0.22	0.06	0.21	0.01	0.01	0.19
**S metabolism**	Adenylylsulfate reductase	<0.01	<0.01	0.01	0.94	0.99	0.95	0.44	0.21	0.43
	Sulfur assimilation	0.16	0.01	0.06	0.05	0.16	0.49	0.49	<0.01	0.54
	Sulfite reduction	<0.01	<0.01	<0.01	0.53	0.08	0.96	0.45	0.71	<0.01
	Sulfide oxidation	<0.01	<0.01	0.01	0.16	0.2	0.06	0.43	0.25	0.45

[+] Abbreviation of environmental attributes: R_h, heterotrophic respiration; R_t, soil total respiration; Q_{10}, temperature sensitivity of heterotrophic respiration.

Supplementary Table 4. The enzyme/protein encoded by biogeochemical cycling genes shown in Fig. 2, Supplementary Figures 9 and 10, and Supplementary Tables 2 and 3.

Gene category	Subcategory	Gene name	Enzyme/protein encoded
C degradation	Glyoxylate cycle	*AceA*	Isocitrate lyase
	Glyoxylate cycle	*AceB*	Malate synthase A
	Starch	glucoamylase	Glucoamylase
	Starch	*cda*	Cyclomaltodextrinase
	Starch	*amyA*	Alpha-amylase
	Starch	*amyX*	Pullulanase
	Starch	*nplT*	Neopullulanase
	Starch	*apu*	Amylopullulanase
	Starch	isopullulanase	Isopullulanase
	Starch	*pula*	Pullulanase, extracellular
	Hemicellulose	xylanase	Xylanase
	Hemicellulose	mannanase	Beta-mannanase
	Hemicellulose	*xyla*	Xylose isomerase
	Hemicellulose	*ara*	Arabinofuranosidase
	Pectin	pectinase	Pectinase
	Pectin	pectin lyase	Pectin lyase
	Pectin	*Pg*	Polygalacturonase
	Pectin	*pel_Cdeg*	Pectin lyase
	Pectin	*rgh*	Rhamnogalacturonase
	Pectin	*pme*	Pectinesterase
	Pectin	exopolygalacturonase	Exopolygalacturonase
	Pectin	*RgaE*	Lipolytic enzyme
	Pectin	*rgl*	Polysaccharide lyase
	Pectin	pectate lyase	Pectate lyase
	Pectin	endopolygalacturonase	Endopolygalacturonase
	Cellulose	*axe*	Acetyl xylan esterase
	Cellulose	cellobiase	Cellobiase
	Cellulose	endoglucanase	Endoglucanase
	Cellulose	cellulase	Cellulase
	Cellulose	exoglucanase	Exoglucanase
	Camphor	camdcab	Camphor 5-monooxygenase
	Terpenes	*limeh*	Limonene-1,2-epoxide hydrolase
	Terpenes	*lmo*	Limonene 1,2-monooxygenase
	Terpenes	*cdh*	Carveol dehydrogenase
	Cutin	cutinase	Cutinase
	Chitin	acetylglucosaminidase	Acetylglucosaminidase
	Chitin	chitin deacetylase	Chitin deacetylase
	Chitin	chitinase	Chitinase
	Vanillin/Lignin	*vana*	Vanillate monooxygenase
	Vanillin/Lignin	*vdh*	Vanillin dehydrogenase
	Vanillin/Lignin	phenol oxidase	Phenol oxidase
	Vanillin/Lignin	ligninase	Ligninase
	Vanillin/Lignin	*glx*	Glyoxal oxidase

	Vanillin/Lignin	*mnp*	Manganese peroxidase
C fixation	Bacterial Microcompartments	*CsoS2*	Carboxysome
	Calvin cycle	*rubisco*	RuBisCo
	Calvin cycle	FBPase	Fructose-1 6-bisphosphatase
	Calvin cycle	*PRK*	Phosphoribulokinase
	Reductive acetyl CCoA	*codh*	Carbon monoxide dehydrogenase
	Reductive acetyl CCoA	*fthfs*	Tetrahydrofolate formylase
	Multiple systems	*pcc*	Propionyl-CoA carboxylase
	reductive tricarboxylic acid cycle	*aclb*	ATP citrate lyase
	reductive tricarboxylic acid cycle	*mdh*	Malate dehydrogenase
N cycling	Ammonification	*gdh*	Glutamate dehydrogenase
	Ammonification	*urec*	Urease
	Anammox	*hzsa*	Hydrazine synthase
	Anammox	*hzo*	Hydrazine oxidoreductase
	Assimilatory N reduction	*narb*	Nitrate reductase
	Assimilatory N reduction	*NiR*	Nitrite reductase
	Assimilatory N reduction	*nira*	Ferredoxin-nitrite reductase
	Assimilatory N reduction	*nirb*	Nitrite reductase
	Assimilatory N reduction	*nasa*	Assimilatory nitrate reductase
	Denitrification	*norb*	Nitric-oxide reductase
	Denitrification	*nirk*	Copper containing nitrite reductase
	Denitrification	*nirs*	Cytochrome cd1 nitrite reductase
	Denitrification	*cnorB*	Nitric oxide reductase
	Denitrification	*nosz*	Nitrous oxide reductase
	Denitrification	*narg*	Respiratory nitrate reductase
	Dissimilatory N reduction	*nrfa*	Ammonia-forming nitrate reductase
	Dissimilatory N reduction	*napa*	Periplasmic nitrate reductase
	Nitrification	*amoa*	Ammonia monooxygenase
	Nitrification	*hao*	Hydroxylamine oxidoreductase
	Nitrogen fixation	*nifh*	Dinitrogenase
P utilization	Phosphorus utilization	phytase	Phytase
	Phosphorus utilization	*ppx*	Exopolyphosphatase
	Phosphorus utilization	*ppk*	Polyphosphate kinase
S metabolism	Adenylylsulfate reductase	*APS_AprA*	Adenylylsulfate reductase
	Adenylylsulfate reductase	*AprA*	Adenylylsulfate reductase
	Adenylylsulfate reductase	*APS_AprB*	Adenylylsulfate reductase
	Sulfur assimilation	cysteine_synthase	Cysteine synthase
	Sulfur assimilation	ATP_sulphurylase	ATP sulphurylase
	Sulfur assimilation	PAPS_reductase	Phosphoadenosine phosphosulfate reductase
	Sulfite reduction	*cysI*	Sulfite reductase
	Sulfite reduction	*cysJ*	Sulfite reductase
	Sulfide oxidation	*sqr*	Sulfide-quinone reductase
	sulfite reduction	*dsrb*	Dissimilatory sulfite reductase
	sulfite reduction	*Sir*	Sulfite reductase
	sulfite reduction	*dsra*	Dissimilatory sulfite reductase
	Sulfur Oxidation	*sox*	Sulfur oxidation cycle enzymes

Supplementary Table 5. Response functions of soil pH, temperature and moisture in MEND model.

Function description	Equation	Eq#
Reaction rate (v) at a specific soil water potential (ψ), soil temperature (T), and soil pH (pH)	$v = v_0 \cdot f(\psi) \cdot f(T) \cdot f(pH)$	(E1)
Response function of soil pH	$f(pH) = \exp\left[-\left(\frac{pH - pH_{\mathrm{opt}}}{pH_{\mathrm{sen}}}\right)^2\right]$	(E2)
Temperature sensitivity of carbon use efficiency (Y_{g})	$Y_{\mathrm{g}}(T) = Y_{\mathrm{g}}(T_{\mathrm{ref}}) - k_{Y\mathrm{g}} \cdot (T - T_{\mathrm{ref}})$	(E3)
Arrhenius equation or Q_{10} method to simulate simulate the response of other parameters to changes in temperature	$f(T) = \exp\left[-\frac{Ea}{R}\left(\frac{1}{T} - \frac{1}{T_{\mathrm{ref}}}\right)\right]$	(E4)
	$f(T) = Q_{10}^{\frac{T - T_{\mathrm{ref}}}{10}}$	(E5)
	$Q_{10} = \exp\left[\frac{Ea}{R \cdot T_{\mathrm{ref}}} \cdot \frac{10}{T}\right]$	(E6)
Soil moisture response function for SOM decomposition by oxidative enzymes	$f_{\mathrm{lig}}(\psi) = \begin{cases} 0, & \psi \le -10^{2.5} \\ 0.625 - 0.25 \times \log_{10}(-\psi), & -10^{2.5} < \psi \le -10^{1.5} \\ 1, & -10^{1.5} < \psi \le -10^{-2.5} \\ [2.5 + 0.4 \times \log_{10}(-\psi)]/1.5, & -10^{-2.5} < \psi \le -10^{-4} \\ 0.6, & \psi > -10^{-4} \end{cases}$	(E7)
Soil moisture response function for SOM decomposition by hydrolytic enzymes	$f_{\mathrm{cel}}(\psi) = \begin{cases} 0, & \psi \le \psi_{\mathrm{min}} \\ 1 - \left[\frac{\ln(\psi/\psi_{\mathrm{FC}})}{\ln(\psi_{\mathrm{min}}/\psi_{\mathrm{FC}})}\right]^b, & \psi_{\mathrm{min}} < \psi \le \psi_{\mathrm{FC}} \\ 1, & \psi > \psi_{\mathrm{FC}} \end{cases}$	(E8)
Soil moisture response function for microbial mortality, dormancy & resuscitation	$f_{\mathrm{A2D}}(\psi) = \frac{(-\psi)^{\omega}}{(-\psi)^{\omega} + (-\psi_{\mathrm{A2D}})^{\omega}}$	(E9)
	$f_{\mathrm{D2A}}(\psi) = \frac{(-\psi_{\mathrm{D2A}})^{\omega}}{(-\psi)^{\omega} + (-\psi_{\mathrm{D2A}})^{\omega}}$	(E10)

Supplementary Table 6. Soil carbon pools (state variables) in the MEND model.

Soil carbon pool	Abbreviation	Variable name in governing equations
Particulate organic matter decomposed by oxidative enzymes	POM_1	P_1
Particulate organic matter decomposed by hydrolytic enzymes	POM_2	P_2
Mineral-associated organic matter	MOM	M
Dissolved organic matter	DOM	D
Active MOM interacting with DOM	QOM	Q
Active microbial biomass	MBA	BA
Dormant microbial biomass	MBD	BD
Oxidative enzymes decomposing POM_1	EP_1	EP_1
Hydrolytic enzymes decomposing POM_2	EP_2	EP_2
Enzymes decomposing MOM	EM	EM

Supplementary Table 7. Governing equations of each soil carbon pool in the MEND model

Governing Equation	Eq#
$\frac{dP_1}{dt} = I_{P1} + (1 - g_D) \cdot F_{12} - F_1$	(S1)
$\frac{dP_2}{dt} = I_{\mathrm{P2}} - F_2$	(S2)
$\frac{dM}{dt} = (1 - f_D) \cdot (F_1 + F_2) - F_3$	(S3)
$\frac{dQ}{dt} = F_4 - F_5$	(S4)
$\frac{dD}{dt} = I_D + f_D(F_1 + F_2) + g_D \cdot F_{12} + F_3 + (F_{14,\mathrm{EP1}} + F_{14,\mathrm{EP2}} + F_{14,\mathrm{EM}}) - F_6 - (F_4 - F_5)$	(S5)
$\frac{dBA}{\mathrm{dt}} = F_6 - (F_7 - F_8) - (F_9 + F_{10}) - F_{12} - (F_{13,\mathrm{EP1}} + F_{13,\mathrm{EP2}} + F_{13,\mathrm{EM}})$	(S6)
$\frac{dBD}{\mathrm{dt}} = (F_7 - F_8) - F_{11}$	(S7)
$\frac{dEP_1}{dt} = F_{13,\mathrm{EP1}} - F_{14,\mathrm{EP1}}$	(S8)
$\frac{dEP_2}{dt} = F_{13,\mathrm{EP2}} - F_{14,\mathrm{EP2}}$	(S9)
$\frac{dEM}{\mathrm{dt}} = F_{13,\mathrm{EM}} - F_{14,\mathrm{EM}}$	(S10)
$\frac{dCO_2}{dt} = (F_9 + F_{10}) + F_{11}$	(S11)
$\frac{d}{\mathrm{dt}}(P_1 + P_2 + M + Q + D + \mathrm{BA} + \mathrm{BD} + \mathrm{EP}_1 + \mathrm{EP}_2 + \mathrm{EM}) = I_{P1} + I_{P2} + I_D - (F_9 + F_{10} + F_{11})$	(S12)

The state variables (C pools) are described in Table S6; Eq. S11 indicates the total heterotrophic respiration flux and Eq. S12 expresses the overall mass balance of the system. The transformation fluxes are elucidated by Eqs. S13–S26 in Table S8.

Supplementary Table 8. Component fluxes in the MEND model (parameters are described in Table S9)

Flux description	Equation	Eq#
Particulate organic carbon (POC) pool 1 (P_1) decomposition (F_1)	$F_1 = \frac{Vd_{P1} \cdot \mathrm{EP}_1 \cdot P_1}{K_{P1} + P_1}$	(S13)
POC pool 2 (P_2) decomposition	$F_2 = \frac{Vd_{P2} \cdot \mathrm{EP}_2 \cdot P_2}{K_{P2} + P_2}$	(S14)
Mineral-associated organic carbon (MOC, M) decomposition	$F_3 = \frac{Vd_M \cdot \mathrm{EM} \cdot M}{K_M + M}$	(S15)
Adsorption (F_4) and desorption (F_5) between dissolved organic carbon (DOC, D) and adsorbed DOC (QOC, Q)	$F_4 = k_{\mathrm{ads}} \cdot (1 - Q/Q_{\max}) \cdot D$<br>$F_5 = k_{\mathrm{des}} \cdot (Q/Q_{\max})$	(S16)<br>(S17)
DOC (D) uptake by microbes	$F_6 = \frac{1}{Y_{\mathrm{g}}}(V_{\mathrm{g}} + V_{\mathrm{m}})\frac{D \cdot \mathrm{BA}}{K_D + D}$	(S18)
Dormancy (F_7) and reactivation (F_8) between active (MBA) and dormant (MBD) microbial biomass (BA and BD)	$F_7 = [1 - D/(K_D + D)] \cdot V_{\mathrm{m}} \cdot \mathrm{BA}$<br>$F_8 = D/(K_D + D) \cdot V_{\mathrm{m}} \cdot \mathrm{BD}$	(S19)<br>(S20)
MBA (BA) growth respiration (F_9) and maintenance respiration (F_{10})	$F_9 = \left(\frac{1}{Y_g} - 1\right)\frac{V_{\mathrm{g}} \cdot \mathrm{BA} \cdot \mathrm{D}}{K_D + D}$<br>$F_{10} = \left(\frac{1}{Y_{\mathrm{g}}} - 1\right)\frac{V_{\mathrm{m}} \cdot \mathrm{BA} \cdot \mathrm{D}}{K_D + D}$	(S21)<br>(S22)
MBD (BD) maintenance respiration	$F_{11} = \beta \cdot V_{\mathrm{m}} \cdot \mathrm{BD}$	(S23)
MBA (BA) mortality	$F_{12} = \gamma \cdot V_{\mathrm{m}} \cdot \mathrm{BA}$	(S24)
Synthesis of enzymes for P_1 (EP_1, $F_{13,\mathrm{EP1}}$), enzymes for P_2 (EP_2, $F_{13,\mathrm{EP2}}$), and enzymes for M (EM, $F_{13,\mathrm{EM}}$)	$F_{13,\mathrm{EP1}} = P_1/(P_1 + P_2) \cdot p_{\mathrm{EP}} \cdot V_{\mathrm{m}} \cdot \mathrm{BA}$<br>$F_{13,\mathrm{EP2}} = P_2/(P_1 + P_2) \cdot p_{\mathrm{EP}} \cdot V_m \cdot \mathrm{BA}$<br>$F_{13,\mathrm{EM}} = p_{\mathrm{EM}} \cdot V_{\mathrm{m}} \cdot \mathrm{BA}$	(S25)
Turnover of enzymes (EP_1, EP_2, EM)	$F_{14,\mathrm{EP1}} = r_{\mathrm{E}} \cdot \mathrm{EP}_1$<br>$F_{14,\mathrm{EP2}} = r_{\mathrm{E}} \cdot \mathrm{EP}_2$<br>$F_{14,\mathrm{EM}} = r_{\mathrm{E}} \cdot \mathrm{EM}$	(S26)

Notes: Italic symbols like F_i represent component fluxes in equations. Italic symbols P_1, P_2, M, Q, D, BA, BD, EP_1, EP_2, and EM are state variables (soil carbon pools, see Supplementary Table 6) in equations.

Supplementary Table 9. Microbial-ENzyme Decomposition (MEND) model parameters

ID	Parameter	Description	Units	Eq#
1	LF_0	Initial fraction of P_1, $LF_0 = P_1/(P_1+P_2)$	—	
2	r_0	Initial active fraction of microbes, $r_0 =$ BA/(BA+BD)	—	
3	fINP	Scaling factor for litter input rate	—	
4	Vd_{P1}	Maximum specific decomposition rate for P_1	mg C mg^{-1} C h^{-1}	S13
5	Vd_{P2}	Maximum specific decomposition rate for P_2	mg C mg^{-1} C h^{-1}	S14
6	Vd_M	Maximum specific decomposition rate for M	mg C mg^{-1} C h^{-1}	S15
7	K_{P1}	Half-saturation constant for P_1 decomposition	mg C cm^{-3} soil	S13
8	K_{P2}	Half-saturation constant for P_2 decomposition	mg C cm^{-3} soil	S14
9	K_M	Half-saturation constant for M decomposition	mg C cm^{-3} soil	S15
10	Q_{max}	Maximum sorption capacity	mg C cm^{-3} soil	S16
11	K_{ba}	Binding affinity, Sorption rate $k_{ads} = k_{des} \times K_{ba}$	(mg C cm^{-3} soil)$^{-1}$	S16
12	k_{des}	Desorption rate	mg C cm^{-3} soil h^{-1}	S17
13	r_E	Turnover rate of EP_1, EP_2, and EM	mg C mg^{-1} C h^{-1}	S26
14	p_{EP}	[$V_m \times p_{EP}$] is the production rate of EP (EP_1 + EP_2), V_m is the specific maintenance rate for BA	—	S25
15	fp_{EM}	$fp_{EM} = p_{EM}/p_{EP}$, [$V_{mt} \times p_{EM}$] is the production rate of EM	—	S25
16	f_D	Fraction of decomposed P_1 and P_2 allocated to D	—	S3
17	g_D	Fraction of dead BA allocated to D	—	S1
18	V_g	Maximum specific uptake rate of D for growth	mg C mg^{-1} C h^{-1}	S21
19	α	$= V_m / (V_g + V_m)$	—	S22
20	K_D	Half-saturation constant for microbial uptake of D	mg C cm^{-3} soil	S18
21	$Y_g(T_{ref})$	Intrinsic carbon use efficiency at reference temperature (T_{ref})	—	S28
22	k_{Yg}	Slope for Y_g dependence of temperature	1/°C	S28
23	Q_{10}	Q_{10} for temperature response function	—	S28
24	γ	Max microbial mortality rate = $V_m \times \gamma$	—	S24
25	β	Ratio of dormant maintenance rate to V_m	—	S23
26	ψ_{A2D}	Soil water potential (SWP) threshold for microbial dormancy; both ψ_{A2D} & $\psi_{D2A} < 0$	−MPa	S30
27	τ	$\psi_{D2A} = \psi_{A2D} \times \tau$, ψ_{D2A} is the SWP threshold for microbial resuscitation	—	S30
28	ω	Exponential in SWP function for microbial dormancy or resuscitation	—	S30

Notes: The column "Eq#" lists the major equation # (see Supplementary Table 7 and 8) in which each parameter is used.

Supplementary Table 10. Objective functions used for different response variables in the MEND model parameterization.

Response Variable	Description of Response Variable	Objective Function for Each Response Variable	
		**Control**	**Warming**
R_h	Heterotrophic Respiration	R^2 between Simulated R_h and Observed R_h	R^2 between Simulated R_h and Observed R_h
MBC	Microbial Biomass Carbon	MARE < 20%<br>MBC_mean = 0.025 mg C cm^{-3}<br>(MBC = 2% SOC)<br>MBC_mean_simulated = 0.02	MARE < 5%<br>MBC_mean = 0.02*0.84 = 0.017 mg C cm^{-3}
EnzCo	Oxidative Enzyme Concentration (EnzC)	Correlation (r) between Simulated EnzC and Observed gene abundance (DNA concentration × relative abundance)	MARE between Simulated EnzC and Expected EnzC<br>Expected EnzC = Simulated EnzC at Control × RR
EnzCh	Hydrolytic Enzyme Concentration	Correlation (r) between Simulated EnzC and Observed gene abundance	MARE between Simulated EnzC and Expected EnzC

Notes: RR is the response ratio of gene abundance under warming to that under control. R^2 denotes the coefficient of determination, MARE is the mean absolute relative error, see Methods Eqs. 3–4.

Supplementary Table 11. Activation energy (Ea: kJ mol^{-1}) and Q_{10} values* for cellulases and ligninases

ID	Category	Enzyme	Ea	Q_{10}	Reference
1	Cellulases	β-glucosidase	30.8	1.52	Eivazi and Tabatabai, 1988[1]
2	Cellulases	β-glucosidase	25.3	1.41	Deng and Tabatabai, 1994[2]
3	Cellulases	β-glucosidase	53.2	2.05	Chauve et al., 2010[3]
4	Cellulases	β-glucosidase	39.0	1.70	Vila-Real et al., 2010[4]
5	Cellulases	β-glucosidase	39.7	1.71	Han and Srinivasan, 1969[5]
6	Cellulases	β-glucosidase	54.3	2.09	Plant et al., 1988[6]
7	Cellulases	β-glucosidase	59.6	2.24	Patchett et al., 1987[7]
8	Cellulases	β-glucosidase	31.0	1.52	Patchett et al., 1987[7]
9	Cellulases	β-glucosidase	41.0	1.74	Patchett et al., 1987[7]
10	Cellulases	β-glucosidase	29.4	1.49	Patchett et al., 1987[7]
11	Cellulases	β-glucosidase	79.5	2.93	Patchett et al., 1987[7]
12	Cellulases	β-glucosidase	44.3	1.82	Ait et al., 1979[8]
13	Cellulases	β-glucosidase	24.7	1.40	McClaugherty and Linkins, 1990[9]
14	Cellulases	β-glucosidase	61.1	2.29	McClaugherty and Linkins, 1990[9]
15	Cellulases	β-glucosidase	43.1	1.79	McClaugherty and Linkins, 1990[9]
16	Cellulases	β-glucosidase	33.2	1.57	McClaugherty and Linkins, 1990[9]
17	Cellulases	β-glucosidase	41.3	1.75	McClaugherty and Linkins, 1990[9]
18	Cellulases	β-glucosidase	39.3	1.70	McClaugherty and Linkins, 1990[9]
19	Cellulases	β-glucosidase	57.0	2.16	Rajoka et al., 2004[10]
20	Cellulases	β-glucosidase	15.0	1.23	Yague and Estevez, 1988[11]
21	Cellulases	β-glucosidase	52.0	2.02	Rajoka et al., 2006[12]
22	Cellulases	β-glucosidase	46.0	1.86	Calsavara et al., 2001[13]
23	Cellulases	β-glucosidase	30.1	1.50	Li et al., 1965[14]
24	Cellulases	Cellobiohydrolase	22.2	1.35	Maguire, 1977[15]
25	Cellulases	Cellobiohydrolase	79.4	2.93	Saharay et al., 2010[16]
26	Cellulases	Cellobiohydrolase	13.8	1.21	Rouau and Odier, 1986[17]
27	Cellulases	Cellobiohydrolase	25.9	1.42	Nikolova et al., 1997[18]
28	Cellulases	Cellobiohydrolase	17.5	1.27	Banka et al., 1998[19]
29	Cellulases	Cellobiohydrolase	52.0	2.02	Eriksen and Goksoyr, 1977[20]
30	Cellulases	Cellobiohydrolase	14.7	1.22	Eriksen and Goksoyr, 1977[20]
31	Cellulases	Endo-glucanase	26.1	1.42	Eriksen and Goksoyr, 1977[20]
32	Cellulases	Endo-glucanase	47.2	1.89	Eriksen and Goksoyr, 1977[20]
33	Cellulases	Endo-glucanase	22.8	1.36	Onyike et al., 2008[21]
34	Cellulases	Endo-glucanase	45.0	1.84	Petre et al., 1986[22]
35	Cellulases	Endo-glucanase	26.9	1.44	Warner et al., 2010[23]
36	Cellulases	Endo-glucanase	3.3	1.05	Javed et al., 2008[24]
37	Cellulases	Endo-glucanase	51.0	1.99	Saqib et al., 2010[25]
38	Cellulases	Endo-glucanase	32.7	1.56	Saqib et al., 2010[25]
39	Cellulases	Endo-glucanase	36.2	1.63	Jabbar et al., 2008[26]
40	Cellulases	Endo-glucanase	35.5	1.62	Perez-Avalos et al., 2008[27]

41	Cellulases	Endo-glucanase	35.5	1.62	Siddiqui et al., 2000[28]
42	Cellulases	Endo-glucanase	38.9	1.69	Melnik et al 1999[29]
43	Cellulases	Endo-glucanase	43.9	1.81	McClaugherty and Linkins, 1990[9]
44	Cellulases	Endo-glucanase	37.6	1.66	McClaugherty and Linkins, 1990[9]
45	Cellulases	Endo-glucanase	39.1	1.70	McClaugherty and Linkins, 1990[9]
46	Cellulases	Endo-glucanase	47.7	1.91	McClaugherty and Linkins, 1990[9]
47	Cellulases	Endo-glucanase	53.6	2.07	McClaugherty and Linkins, 1990[9]
48	Cellulases	Endo-glucanase	31.7	1.54	McClaugherty and Linkins, 1990[9]
49	Cellulases	Endo-glucanase	34.0	1.58	McClaugherty and Linkins, 1990[9]
50	Cellulases	Endo-glucanase	31.3	1.53	McClaugherty and Linkins, 1990[9]
51	Cellulases	Endo-glucanase	23.0	1.37	Hong et al., 1986[30]
52	Cellulases	Endo-glucanase	26.8	1.44	Li et al., 1965[14]
53	Cellulases	Endo-glucanase	21.0	1.33	Paljevac et al., 2007[31]
79	Cellulases	Endo-glucanase	48.6	1.93	Trasar-Cepeda et al. 2007[32]
80	Cellulases	α-glucosidase	38.7	1.69	Stone et al. 2012[33]
81	Cellulases	β-glucosidase	41.5	1.75	Stone et al. 2012[33]
82	Cellulases	β-xylosidase	46.8	1.88	Stone et al. 2012[33]
83	Cellulases	Cellobiohydrolase	52.8	2.04	Stone et al. 2012[33]
84	Cellulases	β-glucosidase	28.6	1.47	Trasar-Cepeda et al. 2007[32]
85	Cellulases	Exocellulase	44.8	1.83	McClaugherty & Linkins 1990[9]
86	Cellulases	Endocellulase	50.4	1.98	McClaugherty & Linkins 1990[9]
87	Cellulases	β-glucosidase	56.3	2.14	Kahkonen et al. 2001[34]
88	Cellulases	β-glucosidase	61.8	2.31	Davidson et al. 2012[35]
54	Ligninases	Peroxidase	97.5	3.74	Chisari et al., 2007[36]
55	Ligninases	Peroxidase	57.8	2.19	Chisari et al., 2007[36]
56	Ligninases	Peroxidase	60.0	2.25	Di Nardo et al., 2004[37]
57	Ligninases	Peroxidase	86.3	3.22	Chisari et al., 2008[38]
58	Ligninases	Peroxidase	66.9	2.47	Padiglia et al., 1995[39]
59	Ligninases	Peroxidase	58.5	2.21	Floris et al., 1982[40]
60	Ligninases	Peroxidase	17.2	1.26	McClaugherty and Linkins, 1990[9]
61	Ligninases	Peroxidase	58.5	2.21	McClaugherty and Linkins, 1990[9]
62	Ligninases	Peroxidase	37.1	1.65	McClaugherty and Linkins, 1990[9]
63	Ligninases	Peroxidase	33.8	1.58	McClaugherty and Linkins, 1990[9]
64	Ligninases	Peroxidase	36.1	1.63	McClaugherty and Linkins, 1990[9]
65	Ligninases	Peroxidase	52.0	2.02	McClaugherty and Linkins, 1990[9]
66	Ligninases	Peroxidase	30.5	1.51	McClaugherty and Linkins, 1990[9]
67	Ligninases	Peroxidase	51.2	2.00	McClaugherty and Linkins, 1990[9]
68	Ligninases	Phenol oxidase	54.8	2.10	Niemetz and Gross, 2003[41]
69	Ligninases	Phenol oxidase	57.0	2.16	Aktas et al., 2001[42]
70	Ligninases	Phenol oxidase	55.0	2.11	Di Nardo et al., 2004[37]
71	Ligninases	Phenol oxidase	44	1.81	McClaugherty and Linkins, 1990[9]
72	Ligninases	Phenol oxidase	56.9	2.16	McClaugherty and Linkins, 1990[43]

73	Ligninases	Phenol oxidase	37.2	1.65	McClaugherty and Linkins, 1990[43]
74	Ligninases	Phenol oxidase	56.6	2.15	McClaugherty and Linkins, 1990[43]
75	Ligninases	Phenol oxidase	76.3	2.81	McClaugherty and Linkins, 1990[43]
76	Ligninases	Phenol oxidase	52.9	2.05	McClaugherty and Linkins, 1990[43]
77	Ligninases	Phenol oxidase	57	2.16	McClaugherty and Linkins, 1990[43]
78	Ligninases	Phenol oxidase	42.2	1.77	Sutay Kocabas et al., 2008[44]
89	Ligninases	Phenol oxidase	42.3	1.77	Kocabas et al. 2008[44]
90	Ligninases	Phenol oxidase	44.8	1.83	Zhang et al. 2008[45]
91	Ligninases	Phenol oxidase	22.3	1.35	Valtcheva et al. 2003[46]
92	Ligninases	Phenol oxidase	12.4	1.18	Lo et al. 2001[47]
93	Ligninases	Manganese Peroxidaseoxidase	51.9	2.02	Acevedo et al. 2010[48]
94	Ligninases	Manganese Peroxidaseoxidase	34.4	1.59	Acevedo et al. 2010[48]
95	Ligninases	Phenol oxidase	32.5	1.55	Davidson et al. 2012[35]
96	Ligninases	Phenol oxidase	23	1.37	Annuar et al. 2009[49]

* Q_{10} values are calculated from Ea with a temperature increase from 20 °C to 30 °C.

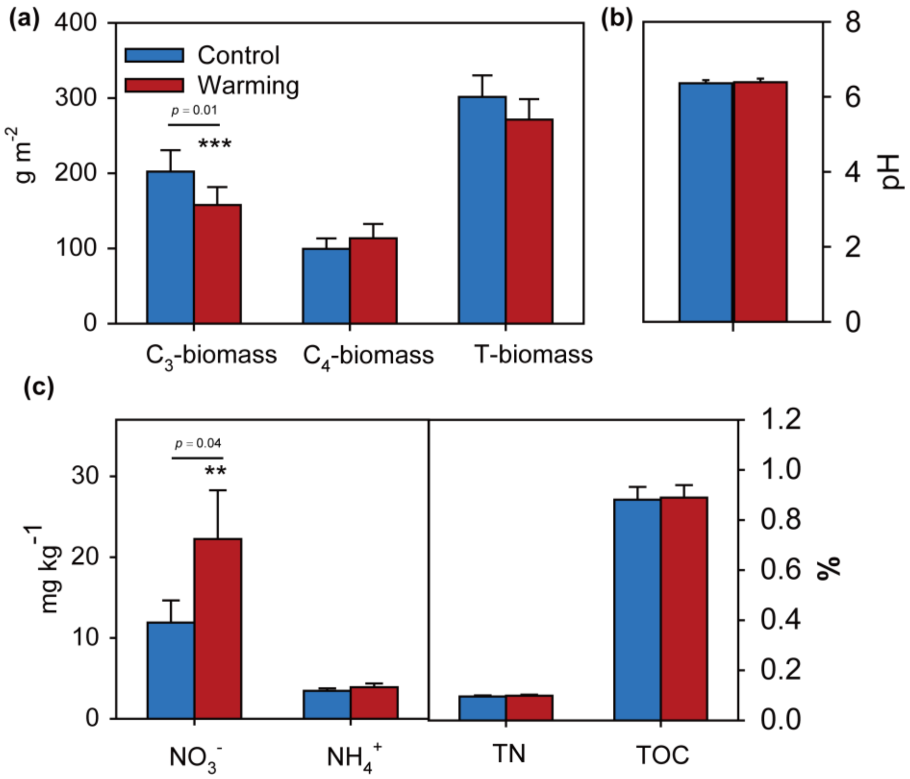

Supplementary Figure 1. Warming effects on plant and soil variables. (a) Effects of warming on aboveground plant biomass from C_3, C_4 and total species; (**b**) Soil pH; (**c**) Soil nitrate (NO_3^-), ammonia (NH_4^+), total N (TN) and total organic carbon (TOC) across 7 years. Error bars represent standard error of the mean (n = 4 field plots examined 7 repeated measures from 2010 to 2016). The differences between warming and the control were tested by the two-sided repeated-measures ANOVA, indicated by *** when $p < 0.01$, ** when $p < 0.05$. Source data are provided as a Source Data file.

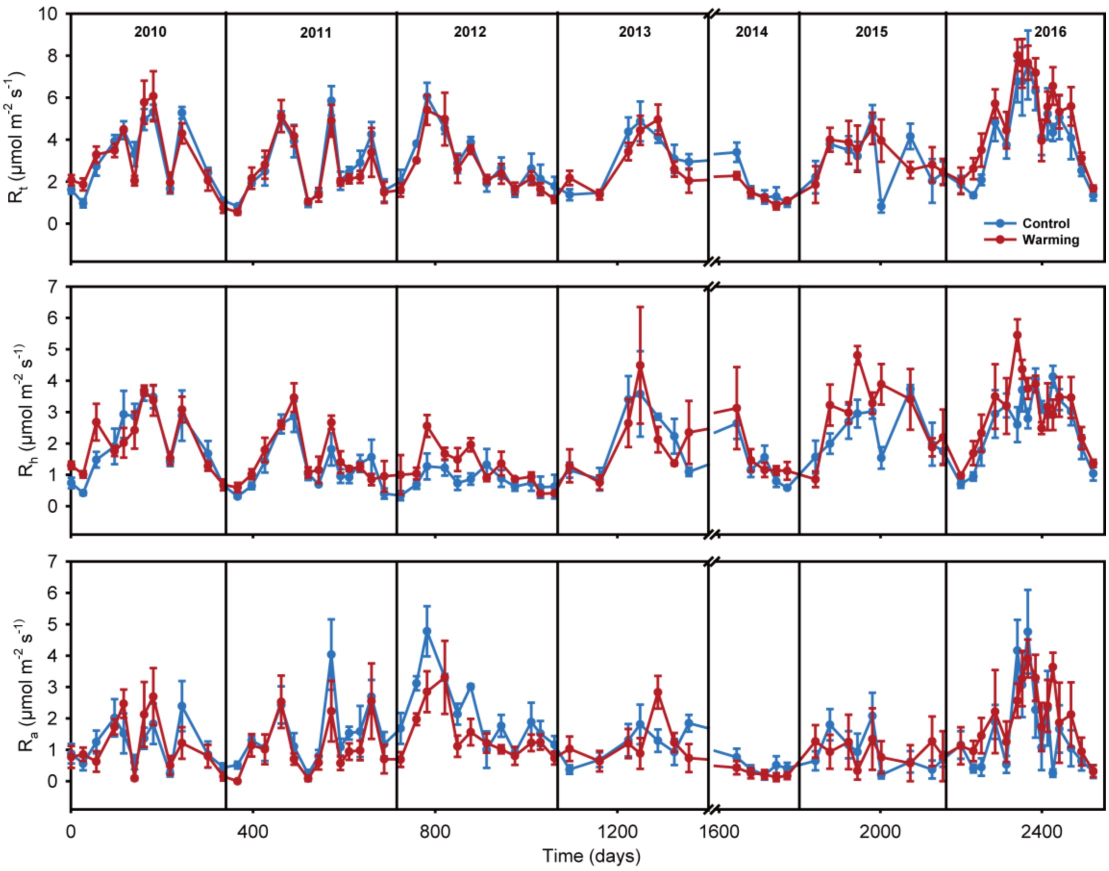

Supplementary Figure 2. Temporal change of soil respiration (R_t), heterotrophic respiration (R_h) and autotrophic respiration (R_a) from 2010 to 2016. The respiration values were displayed as mean ± standard error (*n* = 4 biological field plots).

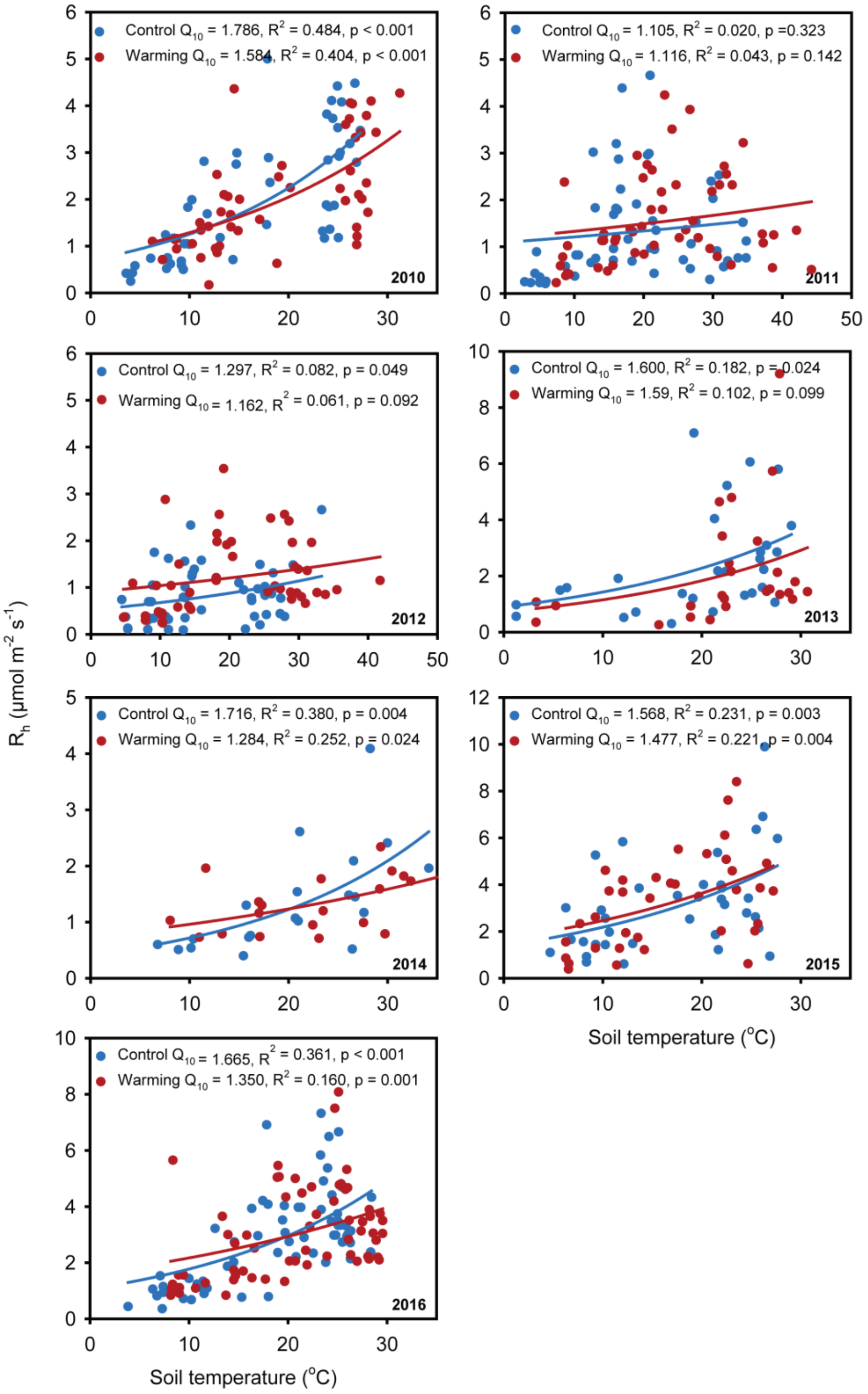

Supplementary Figure 3. Apparent temperature sensitivity of soil heterotrophic respiration (*Q_{10}*). The curve fitting method was used for the control and warming treatments in each year (2010-2016) by exponential growth regression model. Significance was test by analysis of variance (ANOVA).

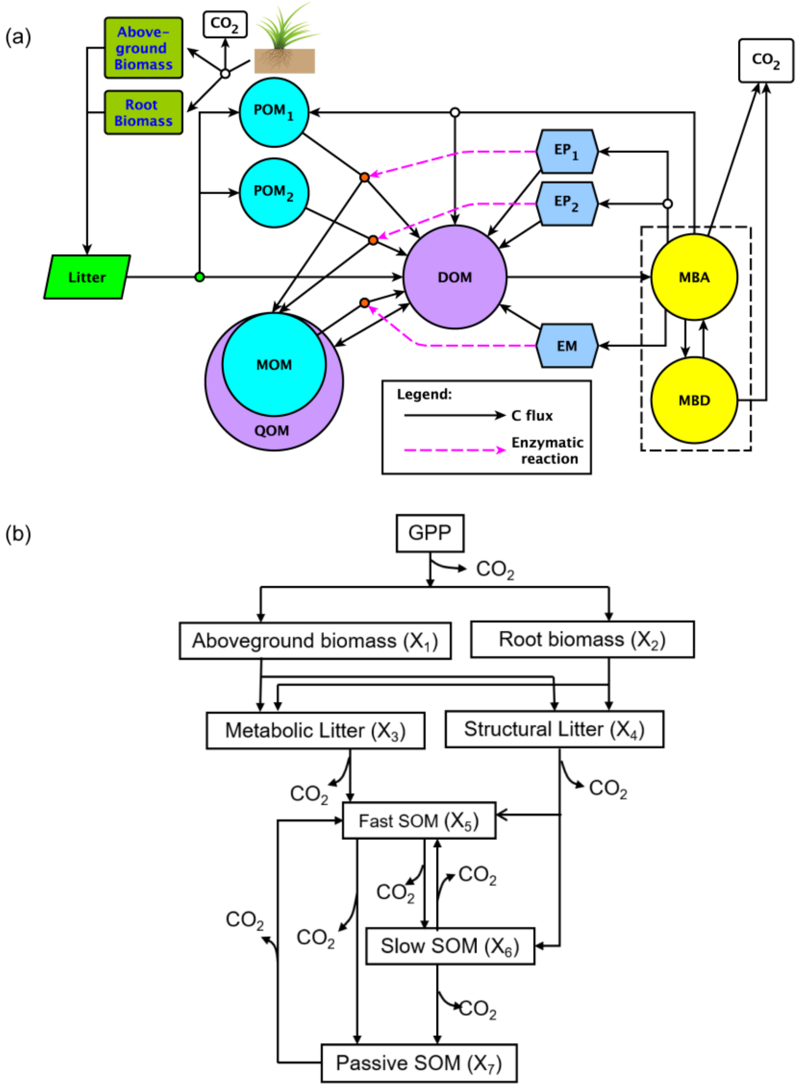

Supplementary Figure 4. Flowcharts of ecosystem models. (a) Microbial-ENzyme Decomposition (MEND) model. Soil organic matter (SOM) pools include: particulate organic matter (POM) (e.g., POM decomposed by oxidative and hydrolytic enzymes, denoted by P_1 and P_2 in the governing equations, respectively), mineral-associated organic matter (MOM, denoted by M), dissolved organic matter (DOM, D), adsorbed phase of DOM (QOM, Q), active and dormant microbes (MBA and MBD, denoted by BA & BD), POM-degraded enzymes (e.g., EP_1 and EP_2 that break down P_1 and P_2, respectively), and MOM-degraded enzymes (EM). **(b)** Terrestrial ECOsystem (TECO) model.

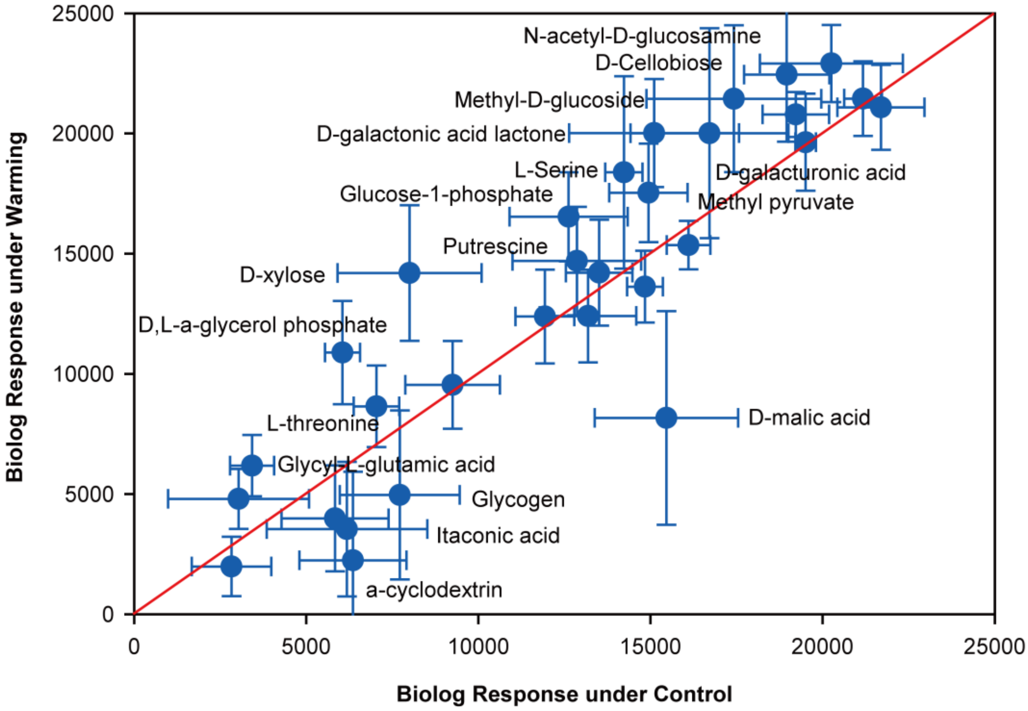

Supplementary Figure 5. A scatterplot of BIOLOG metabolic profiles under warming and control in 2016. Values close to the reference line (red) are in good agreement with the control values. Bi-directional error bars represent standard errors of the mean under control and warming treatments. Values above the reference line have an enhanced ability to utilize that carbon source in the warmed plots, value below have an inhibited ability in the warmed plots.

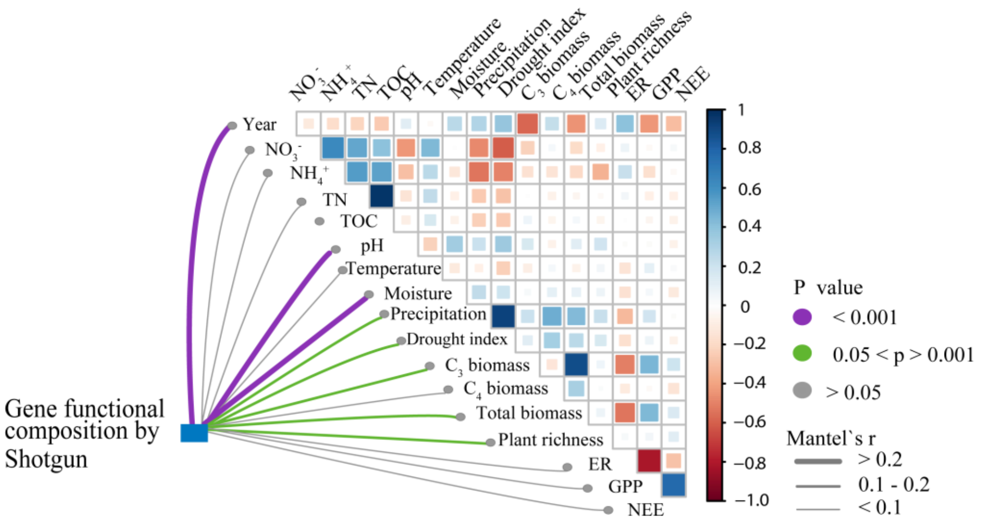

Supplementary Figure 6. Pairwise comparisons of environmental factors with functional community structure based on shotgun sequencing data. The shotgun sequencing data were annotated using EcoFUN-MAP database. A color gradient denotes Pearson's correlation coefficients with functional community structure by partial Mantel tests. Edge width corresponds to the Mantel's *r* statistic for the corresponding distance correlations, and edge color denotes the statistical significance.

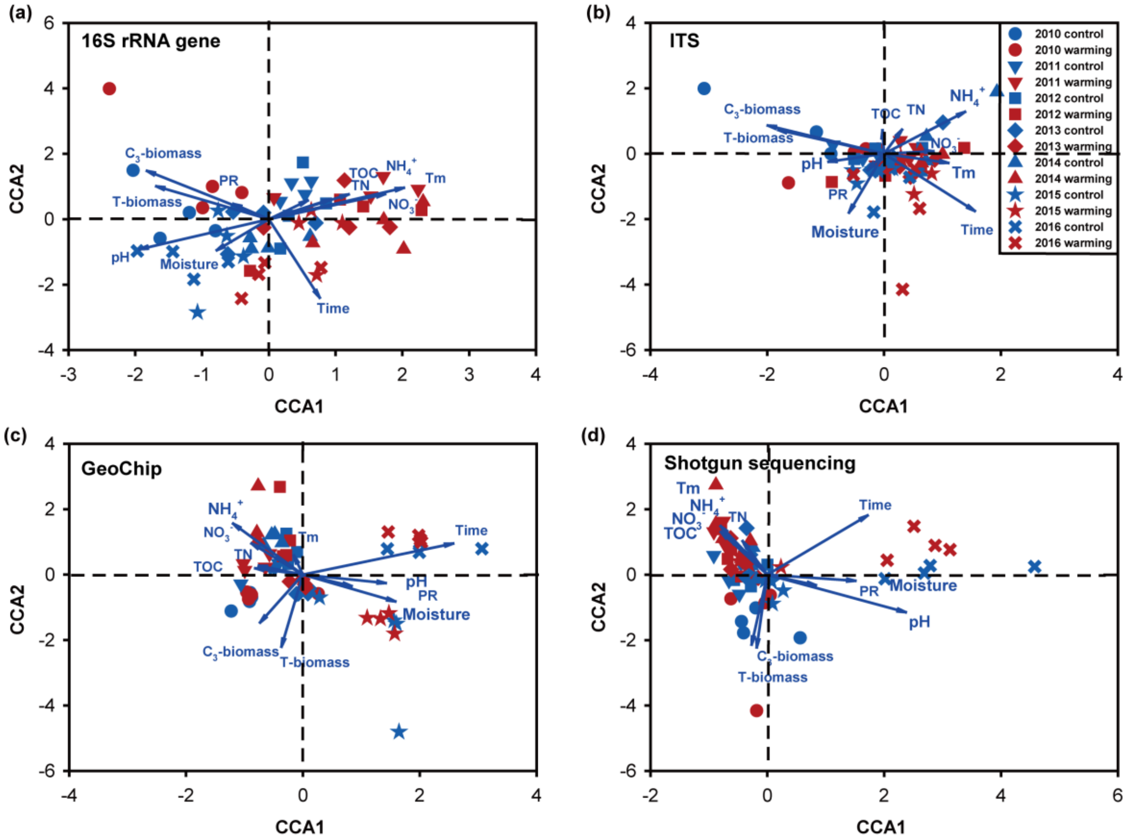

Supplementary Figure 7. Canonical correspondence analyses (CCA) of microbial communities. (a) Bacterial community based on 16S rRNA gene; **(b)** Fungal community based on ITS; **(c)** Functional community based on GeoChip; and **(d)** Functional community based on shotgun metagenomic sequences with EcoFUN-MAP. Phylogenetic and functional structures of microbial communities were significantly shaped by soil related factors: soil temperature (Tm), moisture, soil pH, soil total organic carbon (TOC), total nitrogen (TN), soil nitrate (NO_3^-) and ammonia (NH_4^+) contents; by plant related factors: C_3 and total aboveground plant biomass, and plant richness (PR); and by time.

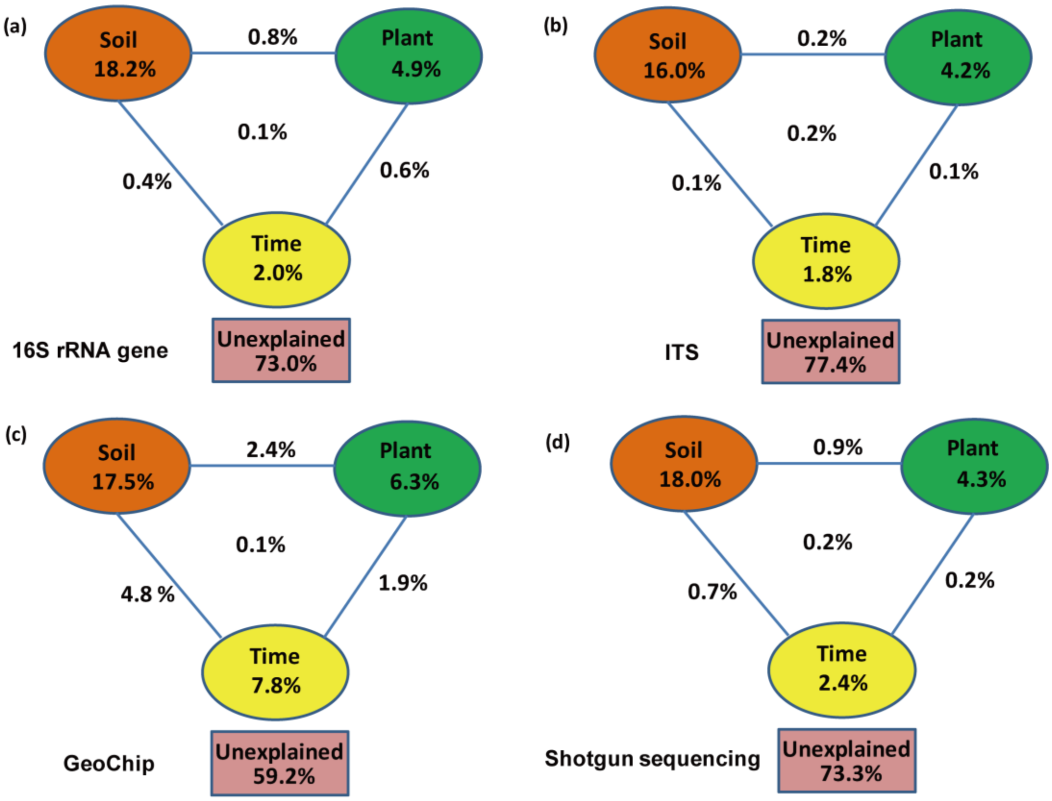

Supplementary Figure 8. CCA-based variation partitioning analysis (VPA) of microbial communities. (a) Bacterial community based on 16S rRNA gene; **(b)** Fungal community based on ITS; **(c)** Functional community based on GeoChip; and **(d)** Functional community based on shotgun metagenomic sequences based on EcoFUN-MAP. The relative proportions of bacterial community variations that can be explained by different types of environmental factors including soil related factors: soil temperature (Tm), moisture, soil pH, soil total organic carbon (TOC), total nitrogen (TN), soil nitrate (NO_3^-) and ammonia (NH_4^+) contents; plant related factors: C_3 and total aboveground plant biomass, and plant richness (PR); and time. The unexplained variations are either due to unmeasured environmental variables and/or stochastic factors.

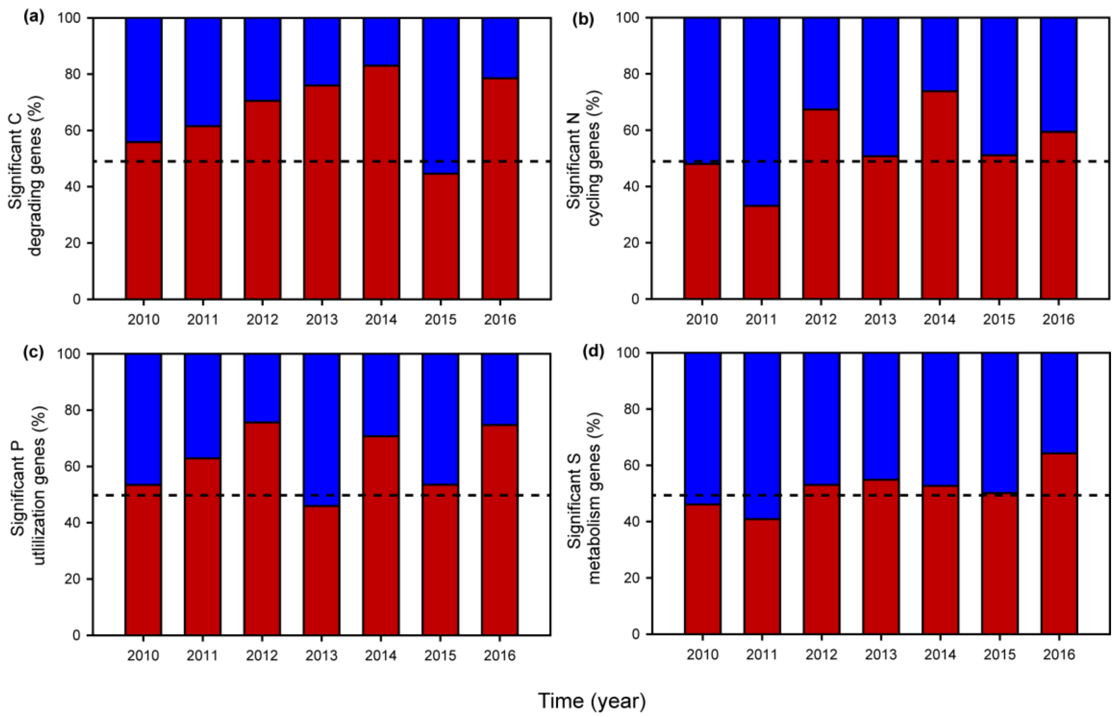

Supplementary Figure 9. Signficantly changed genes involoved in C degradation (a), N cycling (b), P utilization (c) and S metabolism (d) by warming according to GeoChip data. Significance is based on response ratio of each gene with 95% confidence intervals of abundance differences between warmed and control treatments. Dash line represents that the abundance of warming-stimulated (red) genes are in good agreement with the abundance of warming-inhibited (blue) genes. The genes involved in C degradation, N cycling, P utilization and S metabolism in this plot are listed in Supplementary Table 4.

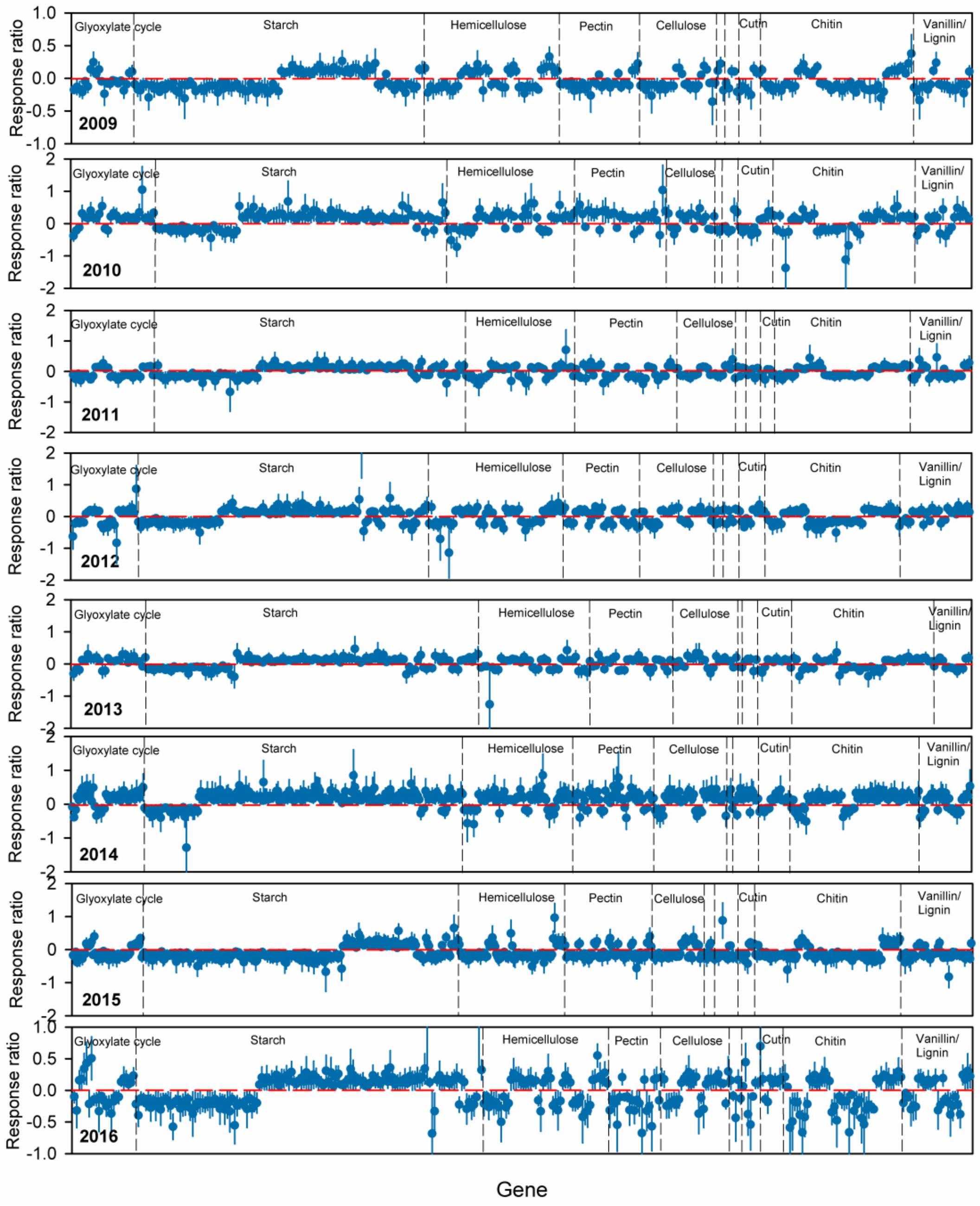

Supplementary Figure 10. Response ratios showing significant changes in abundance of C degradation genes in each year detected by GeoChip. Warming-stimulated C degrading genes were more than warming- inhibited genes in most years. Error bars represented 95% confidence intervals of abundance differences between warmed and control treatments. The targeted substrates were arranaged in order from labile to recalcitrant C. The full names of the genes in this figure are listed in Supplementary Table 4.

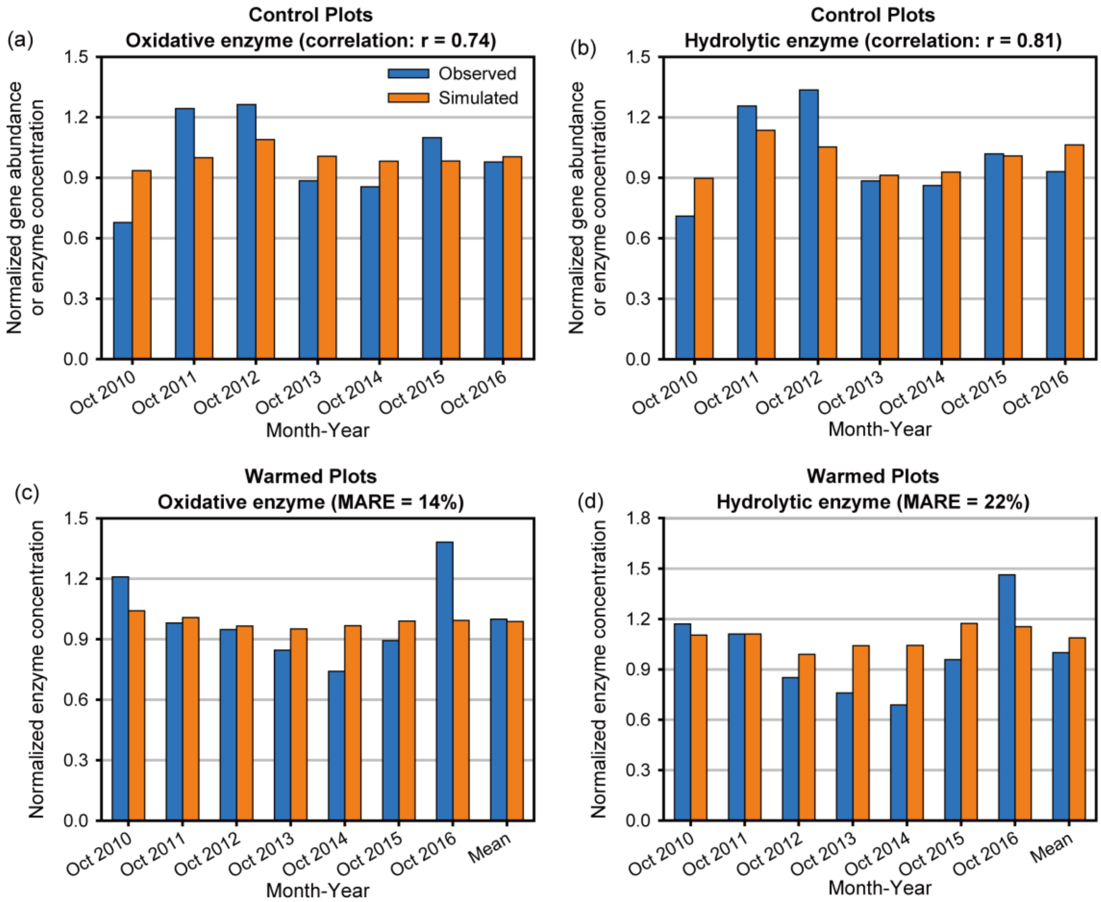

Supplementary Figure 11. MEND modeling performance with gene abundance data. MEND-simulated enzyme concentrations vs. GeoChip gene abundances for (**a**) oxidative enzymes and (**b**) hydrolytic enzymes in the control plot. MEND-simulated enzyme concentrations vs. GeoChip-informed enzyme concentrations for (**c**) oxidative enzymes and (**d**) hydrolytic enzymes in the warmed plot. The model performance for the control plot is quantified by the correlation coefficient (*r*), as we cannot directly compare the absolute values between GeoChip gene abundances and MEND enzyme concentrations. The model performance for the simulations under warming is evaluated by the Mean Absolute Relative Error (MARE) (see Table S9). Lower MARE value means better performance. All data are normalized by their respective mean values.

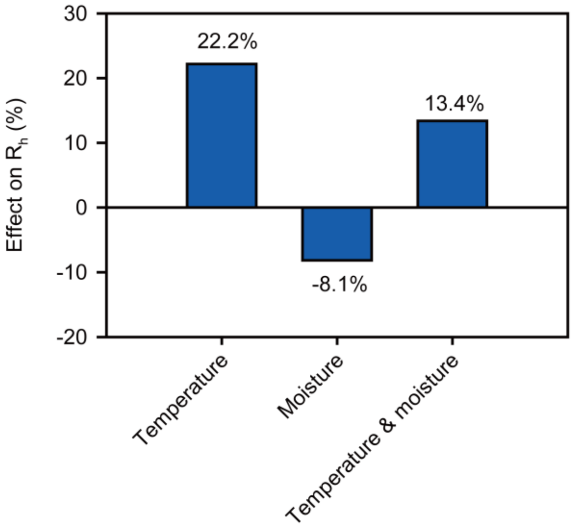

Supplementary Figure 12 The impact of changing temperature vs. changing moisture on soil R_h estimated by the gMEND model. The negative effect on R_h due to slightly drier soil under warming treatment was considerable, but it was completely shifted by the significant positive effect by increasing soil temperature.

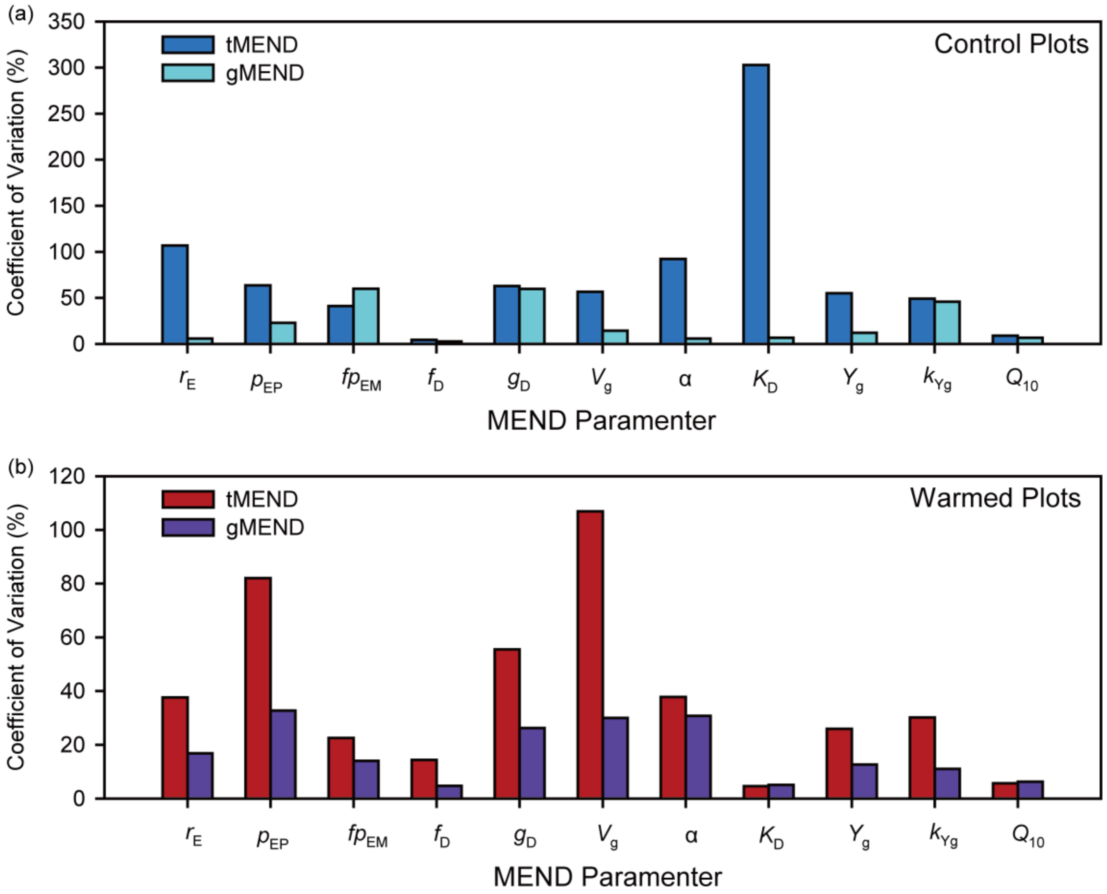

Supplementary Figure 13. The MEND model parameter uncertainty was quantified by the Coefficient of Variation (CV) in the (a) Control and (b) Warmed plot. The tMEND refers to the traditional MEND model parameterization without gene abundances data. The gMEND denotes the improved MEND parameterization with gene abundances. The 11 model parameters are r_E: enzyme turnover rate; p_{EP} and fp_{EM}: two coefficients controlling enzyme production rates; f_D: fraction of decomposed particulate organic matter (POM) entering dissolved organic matter (DOM) pool; g_D: fraction of dead microbe entering DOM pool; V_g: maximum specific growth rate for microbe; α: a coefficient relating specific microbial maintenance rate (V_m) to growth rate ($\alpha = V_m / (V_g + V_m)$); K_D: half-saturation constant for microbial uptake of DOM; Y_g: carbon use efficiency at reference temperature; k_{Yg}: temperature sensitivity of Y_g; Q_{10}: temperature sensitivity of enzyme-catalyzed soil organic matter decomposition. See Table S9 for detailed description of all model parameters.

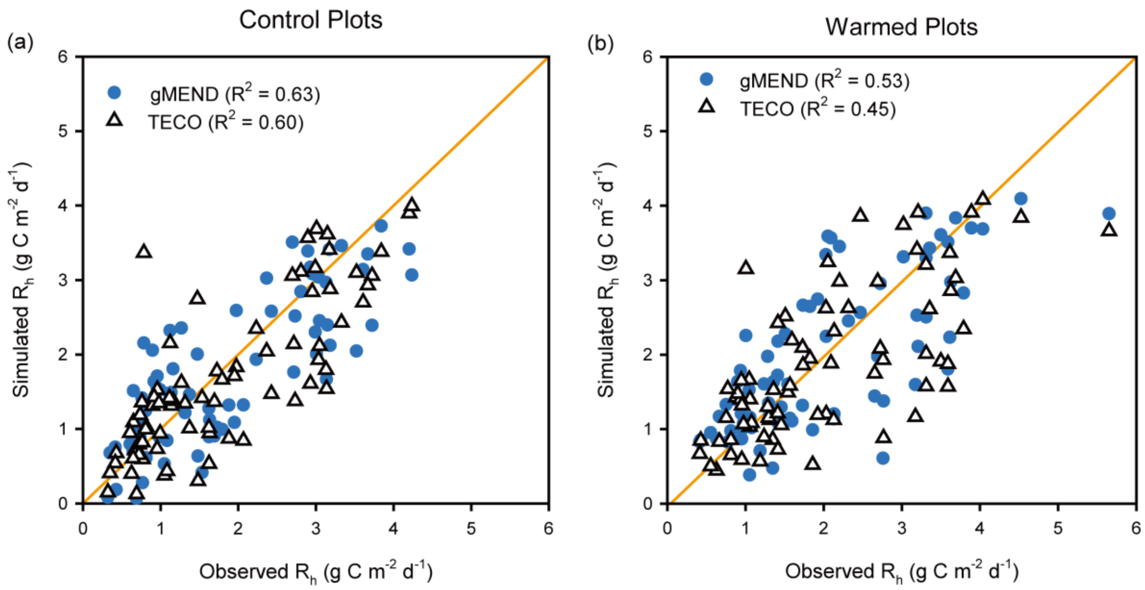

Supplementary Figure 14. **Improvement of model performance with gMEND compared to the non-microbial model TECO**. (**a**) Control plots. (**b**) Warmed plots.

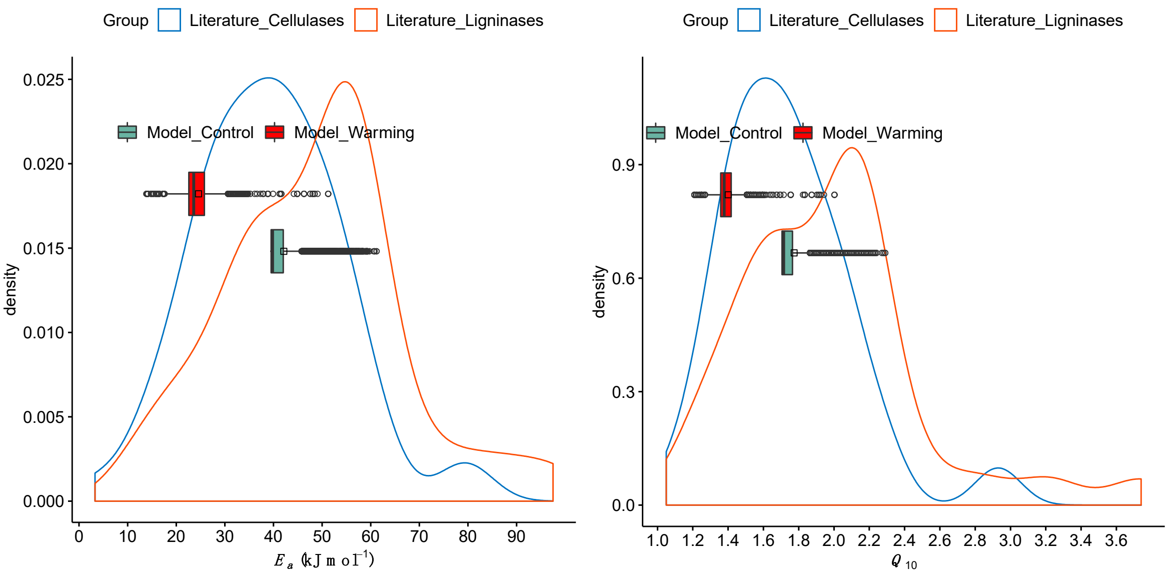

Supplementary Figure 15 Activation energy (Ea) and corresponding Q_{10} values from literature and our model estimates. Literature-Ea values are pooled data from major ligninases and cellulases catalyzing the decomposition of soil organic carbon. Literature-Q_{10} values (n = 63 and 33 for cellulases and ligninases, respectively) are calculated from Ea with a temperature increase from 20 °C to 30 °C. Model-derived Q_{10} values are those under control (n = 7,560) and warming (+3°C, n = 2,095) treatments. Model-Ea values are calcualted from Q_{10} with a temperature increase from 20 °C to 30 °C. Boxplots depict median, first and third quartiles, and full ranges (bounded at 1.5 × interquartile range).

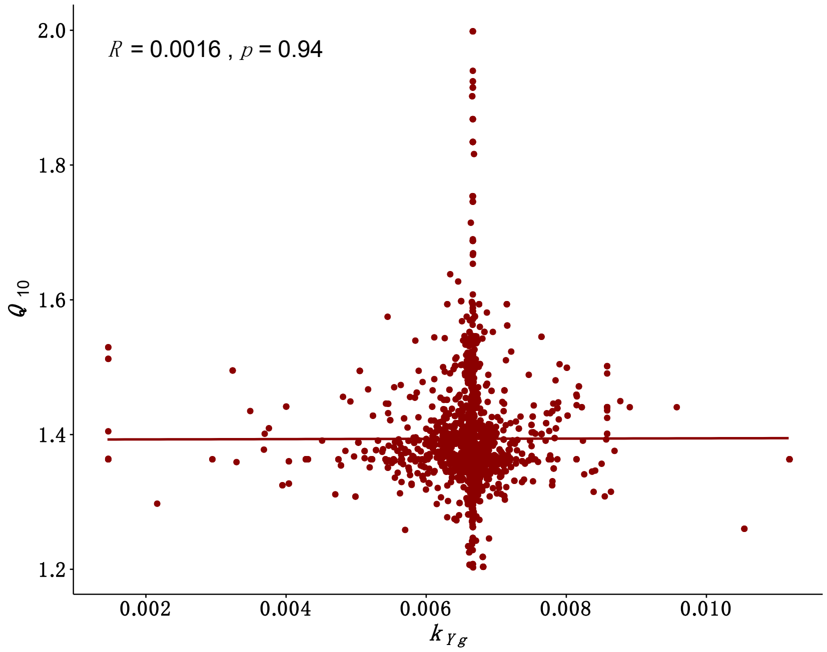

Supplementary Figure 16 Correlation between Q_{10} and k_{Yg} (temperature sensitivity of Y_g). Y_g is the true growth yield, i.e., a proxy for carbon use efficiency (CUE) in the MEND model. The temperature dependence of Y_g on soil temperature (T) is described by $Y_g(T) = Y_g(T_{ref}) - k_{Yg} \cdot (T - T_{ref})$, where $Y_g(T)$ and $Y_g(T_{ref})$ are the Y_g at soil temperture T and T_{ref} (reference temperature), respectively; and k_{Yg} denote the temperature sensitivity of Y_g.

Supplementary References

1 Eivazi, F. & Tabatabai, M. A. Glucosidases and galactosidases in soils. *Soil Biology & Biochemistry* **20**, 601-606 (1988).
2 Deng, S. P. & Tabatabai, M. A. Cellulase activity of soils. *Soil Biology & Biochemistry* **26**, 1347-1354 (1994).
3 Chauve, M. *et al.* Comparative kinetic analysis of two fungal β-glucosidases. *Biotechnology for Biofuels* **3**, 3 (2010).
4 Vila-Real, H., Alfaia, A. J., Phillips, R. S., Calado, A. R. & Ribeiro, M. H. L. Pressure-enhanced activity and stability of α-L-rhamnosidase and β-D-glucosidase activities expressed by naringinase. *Journal of Molecular Catalysis B: Enzymatic* **65**, 102-109 (2010).
5 Han, Y. & Srinivasan, V. Purification and characterization of β-Glucosidase of *Alcaligenes faecalis*. *Journal of bacteriology* **100**, 1355-1363 (1969).
6 Plant, A. R., Oliver, J. E., Patchett, M. L., Daniel, R. M. & Morgan, H. W. Stability and substrate specificity of a β-glucosidase from the thermophilic bacterium Tp8 cloned into *Escherichia coli*. *Archives of Biochemistry and Biophysics* **262**, 181-188 (1988).
7 Patchett, M. L., Daniel, R. M. & Morgan, H. W. Purification and properties of a stable β-glucosidase from an extremely thermophilic anaerobic bacterium. *Biochem. J.* **243**, 779-770 (1987).
8 Ait, N., Creuzet, N. & Cattaneo, J. Properties of β-glucosidase purified from *Clostridium thermocellum*. *Journal of General Microbiology* **128**, 569-577 (1982).
9 McClaugherty, C. & Linkins, A. Temperature responses of enzymes in two forest soils. *Soil Biology and Biochemistry* **22**, 29-33 (1990).
10 Rajoka, M. I., Latif, F., Khan, S. & Shahid, R. Kinetics of improved productivity of β-galactosidase by a cycloheximide-resistant mutant of Kluyveromyces marxianus. *Biotechnology Letters* **26**, 741-746 (2004).
11 Yague, E. & Estevez, M. P. Purification and characterization of a glucosidase from *Evernia prunastri*. *European Journal of Biochemistry* **175**, 627-632 (1988).
12 Rajoka, M. I., Akhtar, M. W., Hanif, A. & Khalid, A. Production and characterization of a highly active cellobiase from *Aspergillus niger* grown in solid state fermentation. *World Journal of Microbiology and Biotechnology* **22**, 991-998 (2006).
13 Calsavara, L. P. V., De Moraes, F. F. & Zanin, G. M. Comparison of catalytic properties of free and immobilized cellobiase Novozym 188. *Applied Biochemistry and Biotechnology* **91**, 615-626 (2001).
14 Li, Y. T. & Shetlar, M. R. GLYCOSIDASES IN THE EARTHWORM, LUMBRICUS TERRESTRIS. *Comparative Biochemistry & Physiology* **14**, 275,IN273,279-278,IN276,279 (1965).
15 Maguire, R. J. Kinetics of hydrolysis of cellulose by β-1,4-glucan cellobiohydrolase of *Trichoderma viride*. *Canadian Journal of Biochemistry* **55**, 644-650 (1977).
16 Saharay, M., Guo, H.-B., Smith, J. C. & Guo, H. in *Computational Modeling in Lignocellulosic Biofuel Production* Vol. 1052 *ACS Symposium Series* Ch. 7, 135-154 (American Chemical Society, 2010).
17 Rouau, X. & Odier, E. Production of extracellular enzyme by the white-rot fungus Dichomitus squalens in cellulose-containing liquid culture. *Enzyme & Microbial Technology* **8**, 22-26 (1986).
18 Nikolova, P. V., Creagh, A. L., Duff, S. J. B. & Haynes, C. A. Thermostability and irreversible activity loss of exoglucanase/xylanase Cex from *Cellulomonas fimi*. *Biochemistry* **36**, 1381-1388 (1997).
19 Banka, D. & Crossley, D. Noise levels of superconducting gravimeters at seismic frequencies. *Geophysical Journal International* **139**, 87-97 (1999).
20 Eriksen, J. & Goksoyr, J. Cellulases from Chaetomium thermophile var. dissitum. *Eur J Biochem* **77**, 445-450 (1977).
21 Onyike, E., Auta, R. & Nok, A. Isolation, partial purification and characterization of endoglucanase (EC. 3.2. 1.4) from *Aspergillus niger* SL 1 using corn cobs as carbon source. *Nigerian Journal of Biochemistry and Molecular Biology* **23**, 1-11 (2008).

22 Pétré, D. *et al.* Purification and properties of the endoglucanase C of *Clostridium thermocellum* produced in *Escherichia coli*. *Biochimie* **68**, 687-695 (1986).
23 Warner, C. D. *et al.* Tertiary structure and characterization of a glycoside hydrolase family 44 endoglucanase from *Clostridium acetobutylicum*. *Applied and Environmental Microbiology* **76**, 338-346 (2010).
24 Javed, M., Rashid, M., Nadeem, H., Riaz, M. & Perveen, R. Catalytic and thermodynamic characterization of Endoglucanase (CMCase) from *Aspergillus oryzae* cmc-1. *Applied Biochemistry and Biotechnology* **157**, 483-497 (2009).
25 Saqib, A. A. N., Hassan, M., Khan, N. F. & Baig, S. Thermostability of crude endoglucanase from *Aspergillus fumigatus* grown under solid state fermentation (SSF) and submerged fermentation (SmF). *Process Biochemistry* **45**, 641-646 (2010).
26 Jabbar, A., Rashid, M. H., Javed, M. R., Perveen, R. & Malana, M. A. Kinetics and thermodynamics of a novel endoglucanase (CMCase) from *Gymnoascella citrina* produced under solid-state condition. *Journal of Industrial Microbiology and Biotechnology* **35**, 515-524 (2008).
27 Pérez-Avalos, O., Sánchez-Herrera, L. M., Salgado, L. M. & Ponce-Noyola, T. A bifunctional endoglucanase/endoxylanase from *Cellulomonas flavigena* with potential use in industrial processes at different pH. *Current Microbiology* **57**, 39-44 (2008).
28 Siddiqui, K. S., Saqib, A. A. N., Rashid, M. H. & Rajoka, M. I. Carboxyl group modification significantly altered the kinetic properties of purified carboxymethylcellulase from *Aspergillus niger*. *Enzyme and Microbial Technology* **27**, 467-474 (2000).
29 Mel'Nik, M. S., Gerner, M. L. & Rabinovich, M. L. A low-molecular-weight endoglucanase from Clostridium thermocellum similar to endoglucanase C. The specificity of effects on synthetic substrates and the amino acid composition. *Прикладная биохимия и микробиология* **35**, 616-623 (1999).
30 Hong, S.-W., Hah, Y.-C., Maeng, P.-J. & Jeong, C.-S. Purification and mode of action of low molecular weight β-1,4-glucan glucanohydrolase from Trichoderma koningii. *Enzyme and Microbial Technology* **8**, 227-235 (1986).
31 Paljevac, M., Primožič, M., Habulin, M., Novak, Z. & Knez, Ž. Hydrolysis of carboxymethyl cellulose catalyzed by cellulase immobilized on silica gels at low and high pressures. *The Journal of Supercritical Fluids* **43**, 74-80 (2007).
32 Trasar-Cepeda, C., Gil-Sotres, F. & Leirós, M. C. Thermodynamic parameters of enzymes in grassland soils from Galicia, NW Spain. *Soil Biology and Biochemistry* **39**, 311-319 (2007).
33 Stone, M. M. *et al.* Temperature sensitivity of soil enzyme kinetics under N-fertilization in two temperate forests. *Global Change Biology* **18**, 1173-1184 (2012).
34 Salkinojasalonen, M. S. Microbial activity of boreal forest soil in a cold climate. *Boreal Environment Research* **6**, 19-28 (2001).
35 Davidson, E. A., Samanta, S., Caramori, S. S. & Savage, K. The Dual Arrhenius and Michaelis–Menten kinetics model for decomposition of soil organic matter at hourly to seasonal time scales. *Global Change Biology* **18**, 371-384 (2012).
36 Chisari, M., Barbagallo, R. N. & Spagna, G. Characterization of Polyphenol Oxidase and Peroxidase and Influence on Browning of Cold Stored Strawberry Fruit. *Journal of Agricultural and Food Chemistry* **55**, 3469-3476 (2007).
37 Di Nardo, C., Cinquegrana, A., Papa, S., Fuggi, A. & Fioretto, A. Laccase and peroxidase isoenzymes during leaf litter decomposition of Quercus ilex in a Mediterranean ecosystem. *Soil Biology and Biochemistry* **36**, 1539-1544 (2004).
38 Chisari, M., Barbagallo, R. N. & Spagna, G. Characterization and Role of Polyphenol Oxidase and Peroxidase in Browning of Fresh-Cut Melon. *Journal of Agricultural & Food Chemistry* **56**, 132-138 (2008).
39 Padiglia, A., Cruciani, E., Pazzaglia, G., Medda, R. & Floris, G. Purification and characterization of Opuntia peroxidase. *Phytochemistry* **38**, 295-297 (1995).

40 Floris, G., Medda, R. & Rinaldi, A. Peroxidase from Ipomoea batatas seedlings: Purification and properties. **23**, 1527-1529 (1984).

41 Niemetz, R. & Gross, G. G. Oxidation of pentagalloylglucose to the ellagitannin, tellimagrandin II, by a phenol oxidase from *Tellima grandiflora* leaves. *Phytochemistry* **62**, 301-306 (2003).

42 Aktas, N. *et al.* Reaction kinetics for laccase-catalyzed polymerization of 1-naphthol. *Bioresource Technology* **80**, 29-36 (2001).

43 Kersten, P. J., Kalyanaraman, B., Hammel, K. E., Reinhammar, B. & Kirk, T. K. Comparison of lignin peroxidase, horseradish peroxidase and laccase in the oxidation of methoxybenzenes. *Biochemical Journal* **268**, 475-480 (1990).

44 Kocabas, D. S., Bakir, U., Phillips, S. E. V., McPherson, M. J. & Ogel, Z. B. Purification, characterization, and identification of a novel bifunctional catalase-phenol oxidase fromScytalidium thermophilum. **79**, 407-415 (2008).

45 Zhang, J., Liu, X., Xu, Z., Chen, H. & Yang, Y. Degradation of chlorophenols catalyzed by laccase. *International Biodeterioration & Biodegradation* **61**, 351-356 (2008).

46 Valtcheva, E., Veleva, S., Radeva, G. & Valtchev, I. Enzyme action of the laccase-mediator system in the pulp delignification process. *Reaction Kinetics and Catalysis Letters* **78**, 183-191 (2003).

47 Lo, S., Ho, Y. & Buswell, J. Effect of phenolic monomers on the production of laccases by the edible mushroom Pleurotus sajor-caju, and partial characterization of a major laccase component. *Mycologia* **93**, 413-421 (2001).

48 Acevedo, F. *et al.* Degradation of polycyclic aromatic hydrocarbons by free and nanoclay-immobilized manganese peroxidase from Anthracophyllum discolor. *Chemosphere* **80**, 271-278 (2010).

49 Annuar, M. S. M., Adnan, S., Vikineswary, S. & Chisti, Y. Kinetics and Energetics of Azo Dye Decolorization by Pycnoporus sanguineus. *Water, Air, and Soil Pollution* **202**, 179-188 (2009).
