# Supplementary material for: Gene-informed decomposition model predicts lower soil carbon loss due to persistent microbial adaptation to warming
Source: Nat Commun. 2020 Sep 29;11:4897. doi: 10.1038/s41467-020-18706-z (PMC7524716; doi:10.1038/s41467-020-18706-z)
Supplement: Supplementary file 3 — Reporting Summary [file 41467_2020_18706_MOESM3_ESM.pdf]

natureresearch

Corresponding author(s): Jizhong Zhou

Last updated by author(s): Aug 3, 2020

Reporting Summary

Nature Research wishes to improve the reproducibility of the work that we publish. This form provides structure for consistency and transparency in reporting. For further information on Nature Research policies, see Authors & Referees and the Editorial Policy Checklist.

Statistics

For all statistical analyses, confirm that the following items are present in the figure legend, table legend, main text, or Methods section.

n/a	Confirmed	
☐	☒	The exact sample size (*n*) for each experimental group/condition, given as a discrete number and unit of measurement
☐	☒	A statement on whether measurements were taken from distinct samples or whether the same sample was measured repeatedly
☐	☒	The statistical test(s) used AND whether they are one- or two-sided<br>*Only common tests should be described solely by name; describe more complex techniques in the Methods section.*
☐	☒	A description of all covariates tested
☐	☒	A description of any assumptions or corrections, such as tests of normality and adjustment for multiple comparisons
☐	☒	A full description of the statistical parameters including central tendency (e.g. means) or other basic estimates (e.g. regression coefficient) AND variation (e.g. standard deviation) or associated estimates of uncertainty (e.g. confidence intervals)
☐	☒	For null hypothesis testing, the test statistic (e.g. *F*, *t*, *r*) with confidence intervals, effect sizes, degrees of freedom and *P* value noted<br>*Give P values as exact values whenever suitable.*
☒	☐	For Bayesian analysis, information on the choice of priors and Markov chain Monte Carlo settings
☐	☒	For hierarchical and complex designs, identification of the appropriate level for tests and full reporting of outcomes
☐	☒	Estimates of effect sizes (e.g. Cohen's *d*, Pearson's *r*), indicating how they were calculated

Our web collection on statistics for biologists contains articles on many of the points above.

Software and code

Policy information about availability of computer code

Data collection	All the software used in current study for data collection are commercial. Daily GPP values were obtained from a corrected 8-day GPP product based on the MODIS GPP (MOD17A2/MOD17A2H).
Data analysis	Raw amplicon sequences were analyzed in the Galaxy sequence analysis pipeline (http://zhoulab5.rccc.ou.edu:8080). OTUs were clustered by UPARSE (2013). Geochip data were analyzed in the Microarray Data Manager on our website (http://ieg.ou.edu/microarray). Shotgun data were analyzed in our EcoFUN-MAP pipeline (http://www.ou.edu/ieg/tools/data-analysis-pipeline.html). Statistical analyses were performed in R version 3.1.1 (www.R-project.org) or available pipelines. Detailed information is provided in the text. The code for modeling analysis are performed in FORTRAN and accessible at https://github.com/wanggangsheng/MENDokw.git. We used the Shuffled Complex Evolution (SCE) algorithm to determine model parameters. We also applied the probabilistic inversion (Markov Chain Monte Carlo) to quantity parameter uncertainties.

For manuscripts utilizing custom algorithms or software that are central to the research but not yet described in published literature, software must be made available to editors/reviewers. We strongly encourage code deposition in a community repository (e.g. GitHub). See the Nature Research guidelines for submitting code & software for further information.

Data

Policy information about availability of data

All manuscripts must include a data availability statement. This statement should provide the following information, where applicable:

- Accession codes, unique identifiers, or web links for publicly available datasets
- A list of figures that have associated raw data
- A description of any restrictions on data availability

DNA sequences of 16S rRNA gene and ITS amplicons were available in NCBI Sequence Read Archive under project no. PRJNA331185. Raw shotgun metagenomic sequences are deposited in the European Nucleotide Archive (http://www.ebi.ac.uk/ena) under study no. PRJNA533082. GeoChip signal intensity data can be accessed through the URL (https://www.ou.edu/ieg/publications/datasets). MEND model output and raw data of soil respirations with soil moisture and

temperature were provided in an excel file as Supplementary Information. All other relevant data are available in Supplementary Information or from the corresponding author upon request.

Field-specific reporting

Please select the one below that is the best fit for your research. If you are not sure, read the appropriate sections before making your selection.

☐ Life sciences ☐ Behavioural & social sciences ☒ Ecological, evolutionary & environmental sciences

For a reference copy of the document with all sections, see nature.com/documents/nr-reporting-summary-flat.pdf

Ecological, evolutionary & environmental sciences study design

All studies must disclose on these points even when the disclosure is negative.

Study description	The study is a long-term manipulated, climate change, field experiment with 4 warmed (+3 °C) and 4 control plots in a block design. The experiment site was established in July 2009.
Research sample	In this study, we primarily focus on the responses of microbial respiration and community functions to warming. To model microbial respiratory responses to climate warming, long-term experiments under more realistic field-settings with time-series microbial data are needed. Therefore, the study includes a total of 56 annual soil surface (0-15cm) samples (4 warmed plots and 4 control plots in each year) from 2010 to 216. The differences of warmed and control treatments represent the effects of long-term experimental warming on ecosystem functions and soil microbial communities.
Sampling strategy	In this study, 8 surface (0-15 cm) soil samples, four from the warmed and four from the control plots, were collected annually at approximately the date of peak plant biomass (September or October) from 2010 to 2016. Three soil cores (2.5 cm diameter x 15 cm deep) were taken using a soil sampler tube in each plot and composited to have enough samples for soil chemistry, microbiology and molecular biology analyses. A total of 56 soil samples were analyzed in this study.
Data collection	All sample collection from the experiment site was performed by authors XG, MY, LYW and the lab technician. Three soil cores (2.5 cm diameter x 15 cm deep) were collected in each field plots using a soil sampler tube and composited to have enough samples for soil chemistry, microbiology and molecular biology analyses. Plant survey, and measurements of ecosystem C fluxes and soil respirations were performed by the lab technician following standard protocols. Soil DNA extraction and PCR were performed by XG, LC and XZ in the University of Oklahoma. GeoChip hybridization and MiSeq sequencing were performed by X.G., X.Z., and R.T. Soil chemical and substrate analyses were performed by the Soil, Water, and Forage Analytical Laboratory at Oklahoma State University. Soil decomposition rate was measured by JF and XG, and BIOLOG analysis was performed by XG. Shotgun sequencing was performed at the Oklahoma Medical Research Foundation's Genomics Core using the Illumina HiSeq 3000 platform
Timing and spatial scale	In this study, We collected soil samples for seven consecutive years. Specifically, a total of 56 annual soil samples was collected annually from 4 warmed and 4 control plots (September or October) from 2010 to 2016 in this long-term warming experiment site (34 59′ N, 97 31′W). The data of ecosystem C fluxes, soil respirations and plant biomass were also collected annually.
Data exclusions	There were no data exclusions.
Reproducibility	16S rRNA gene and ITS amplicons were sequenced by MiSeq platform(Illumina, SanDiego, CA, USA) using a 500-cycle v2 MiSeq reagent cartridge (Illumina).<br>Shotgun sequencing was performed at the Oklahoma Medical Research Foundation's Genomics Core using the Illumina HiSeq 3000 platform with a 2 x 150 bp paired-end kit.<br>GeoChip 5.0M was used for all 56 samples to analyze functional structure of soil microbial community from 2010 to 2016.<br>Statistical analyses of amplicon sequencing data, shotgun sequencing data and GeoChip data showed consistent results.
Randomization	Treatments were set up in a randomized block design.
Blinding	All samples taken were labeled with a single number to track samples during lab processing, but included no information as to the treatment from which it originated.

Did the study involve field work? ☒ Yes ☐ No

Field work, collection and transport

Field conditions	This experimental site was conducted in an old-field tallgrass prairie abandoned from cropping 40 years ago with light grazing until 2008. Ambrosia trifida, Solanum carolinense and Euphorbia dentate belonging to C3 forbs, and Tridens flavus, Sporobolus compositus and Sorghum halapense belonging to C4 grasses are dominant in the site. Annual mean temperature is 16.3 °C and annual precipitation is 914 mm. The soil type of this site is Port–Pulaski–Keokuk complex with 51% of sand, 35% of silt and 13% of clay, which is a well-drained soil that is formed in loamy sediment on flood plains. The soil has a high available water holding capacity (37%), neutral pH and 1.2 g cm-3 bulk density with 1.9% total organic matter and 0.1% total nitrogen (N).
Location	The experimental site is located at the Kessler Atmospheric and Ecological Field Station (KAEFS) in the US Great Plains in McClain County, Oklahoma (34 59′ N, 97 31′W).

Access and import/export	Project and class site use requests were completed for our study. Liability waivers were completed hard copies provided to KAEFS.
Disturbance	Infrared heaters may disturb the grassland ecosystem. To minimize these disturbances, 'dummy' heaters were used in this study.

Reporting for specific materials, systems and methods

We require information from authors about some types of materials, experimental systems and methods used in many studies. Here, indicate whether each material, system or method listed is relevant to your study. If you are not sure if a list item applies to your research, read the appropriate section before selecting a response.

Materials & experimental systems

n/a	Involved in the study
☒	☐ Antibodies
☒	☐ Eukaryotic cell lines
☒	☐ Palaeontology
☒	☐ Animals and other organisms
☒	☐ Human research participants
☒	☐ Clinical data

Methods

n/a	Involved in the study
☒	☐ ChIP-seq
☒	☐ Flow cytometry
☒	☐ MRI-based neuroimaging
